# Short-Term Effect of Temperature Change on Non-Accidental Mortality in Shenzhen, China

**DOI:** 10.3390/ijerph18168760

**Published:** 2021-08-19

**Authors:** Yao Xiao, Chengzhen Meng, Suli Huang, Yanran Duan, Gang Liu, Shuyuan Yu, Ji Peng, Jinquan Cheng, Ping Yin

**Affiliations:** 1 Department of Epidemiology and Biostatistics, School of Public Health, Tongji Medical College, Huazhong University of Science and Technology, 13 Hangkong Rd, Wuhan 430030, China; xiaoyao_@hust.edu.cn (Y.X.); m201975317@hust.edu.cn (C.M.); d201781165@hust.edu.cn (Y.D.); 2 Shenzhen Center for Disease Control and Prevention, 8 Longyuan Rd, Shenzhen 518055, China; huanwei11@wjw.sz.gov.cn (S.H.); jiceng1@wjw.sz.gov.cn (G.L.); huanwei1@wjw.sz.gov.cn (S.Y.); 3 Shenzhen Center for Chronic Disease Control, 2021 Buxin Rd, Shenzhen 518020, China

**Keywords:** diurnal temperature change, temperature change between neighboring days, distributed lag nonlinear model, mortality, climatic effect, interactive effect

## Abstract

Temperature change is an important meteorological indicator reflecting weather stability. This study aimed to examine the effects of ambient temperature change on non-accidental mortality using diurnal temperature change (DTR) and temperature change between neighboring days (TCN) from two perspectives, intra-day and inter-day temperature change, and further, to explore seasonal variations of mortality, identify the susceptible population and investigate the interaction between temperature change and apparent temperature (AT). We collected daily data on cause-specific mortality, air pollutants and meteorological indicators in Shenzhen, China, from 1 January 2013 to 29 December 2017. A Quasi-Poisson generalized linear regression combined with distributed lag non-linear models (DLNMs) were conducted to estimate the effects of season on temperature change-related mortality. In addition, a non-parametric bivariate response surface model was used to explore the interaction between temperature change and AT. The cumulative effect of DTR was a U-shaped curve for non-accidental mortality, whereas the curve for TCN was nearly monotonic. The overall relative risks (RRs) of non-accidental, cardiovascular and respiratory mortality were 1.407 (95% CI: 1.233–1.606), 1.470 (95% CI: 1.220–1.771) and 1.741 (95% CI: 1.157–2.620) from exposure to extreme large DTR (99th) in cold seasons. However, no statistically significant effects were observed in warm seasons. As for TCN, the effects were higher in cold seasons than warm seasons, with the largest RR of 1.611 (95% CI: 1.384–1.876). The elderly and females were more sensitive, and low apparent temperature had a higher effect on temperature change-related non-accidental mortality. Temperature change was positively correlated with an increased risk of non-accidental mortality in Shenzhen. Both female and elderly people are more vulnerable to the potential adverse effects, especially in cold seasons. Low AT may enhance the effects of temperature change.

## 1. Introduction

The Intergovernmental Panel on Climate Change (IPCC) Fifth Assessment Report indicated that the probability of climate-related extremes, such as heat weaves, has more than doubled in some locations, and it predicts that the strength and range will increase in the future [1]. Many human systems experience significant vulnerability and exposure due to current climate variability. In the context of extreme climate change and the frequent occurrence of adverse weather events, many epidemiological studies have found that extreme temperature change may increase the risk of mortality from various diseases [2,3,4,5].

Diurnal temperature change (DTR) and temperature change between neighboring days (TCN) are common indicators used in reflecting intra-day and inter-day temperature fluctuations, respectively. DTR is described as the difference between the maximum and minimum temperature of one day. In addition, TCN is defined as the current day’s mean temperature minus the previous day’s mean temperature. A negative TCN represents a temperature decrease, and a positive TCN represents a temperature increase between neighboring days. Although temperature and temperature change may be largely influenced by climate change, some epidemiologic studies have examined the role of temperature or season as effect modifiers in temperature change-related mortality. They found that both sudden temperature change and extreme temperature have an impact on the human body, disturbing normal physiological immune systems, causing serious heat and cold stress-related illness and increasing mortality [6,7]. Therefore, temperature change is still considered as a significant and independent risk factor for cardiovascular and respiratory diseases in addition to seasonal and temperature effects [2,8].

Several time-series studies have reported the short-time delayed effect of temperature changes on human health. The delayed effect and non-linear exposure–response relationship can be elegantly described with distributed lag non-linear models (DLNMs), which provide a comprehensive picture of the entire exposure process. Previous studies have assessed the relationship between temperature change and mortality in many cities. Consistently, these studies have identified a non-linear relationship and that larger temperature changes increase the risk of non-accidental mortality, including cardiovascular and respiratory mortality. The adverse impact of temperature variability on mortality has been identified, while showing great differences in causes and seasonal patterns [9,10]. A study conducted in nine regions of the United Kingdom showed that a large DTR exhibited greater adverse effect estimates in the hot season, and the DTR-mortality relationship would be significantly modified by season, average temperature and humidity [11]. Another investigation conducted in eight cities of China established a notable association between DTR and mortality with cardiovascular or respiratory causes during cold seasons, where a 1 °C increment in DTR corresponded to a 0.42% increase in total non-accidental mortality [12]. Additionally, a study from 106 communities in the United States proved that the TCN–mortality association varied by region and season [13]. The modification effects of seasons have been inconsistent in previous studies. Therefore, we divided the year into cold and warm seasons to explore the role of the season as an effect modifier in the temperature change–mortality association, to make up for the limitations of previous studies. For the reason for modification to be the change of apparent temperature, a composite indicator of mean temperature, relative humidity and wind speed reflected real physiological experience, apparent temperature may modify the effect of temperature change-related mortality. A potential interaction may exist between temperature change and apparent temperature, but there has been little research into the possible joint effects of temperature change and apparent temperature on non-accidental mortality.

Therefore, this study aimed to investigate the effects of temperature change, including intra-day and inter-day temperature changes, on non-accidental mortality and to identify the modifier effects of age, sex, cause and season, further exploring whether apparent temperature modulates the effect of temperature change on non-accidental mortality.

## 2. Materials and Methods

### 2.1. Study Design and Data

Shenzhen is located in South China and was one of the earliest special economic zones, with a population of 12.53 million at the end of 2017. It has a subtropical oceanic climate, with hot and rainy summers and changeable springs. We collected 65,333 records from the Shenzhen Center for Disease Control and Prevention (CDC) for a total of 1824 days from 1 January 2013 to 29 December 2017. Then, we classified the individual-level data into non-accidental (ICD-10: A00–R99), cardiovascular (ICD-10: I00–I99) and respiratory (ICD-10: J00–J99), according to the International Classification of Diseases, 10th version (ICD-10). In addition, we stratified the mortality data by sex and age (≥65 years and <65 years). We obtained daily meteorological data, including mean, maximum and minimum temperature (°C), relative humidity (RH, %), and wind speed (WS, m/s) from the Shenzhen National Basic Meteorological Station of Shenzhen Meteorological Service Center, which can be considered as representative for the population living in Shenzhen. Air pollutants data, including NO_2_, PM_10_, CO, SO_2_, O_3_ and PM_2.5_ levels, were collected from seven fixed monitoring stations distributed in different districts of Shenzhen. We used average concentrations of seven stations as the daily mean concentrations of air pollutants.

To control the combined effect of temperature and humidity on humans, we calculated a composite indicator, apparent temperature (*AT*, °C), based on mean temperature (°C), relative humidity (%) and wind speed (m/s), using the following formulas [14]:

(1) $$AT=T_{a}+0.33*e-0.70*WS-4.00$$

(2) $$e=RH/100*6.105*\exp(17.27*T_{a}/(237.7+T_{a}))$$

In Equation (1), *AT* (°C) denotes the apparent temperature; *T_a_* (°C) denotes the daily mean temperature; *e* (hPa) denotes the water vapor pressure, which was calculated with Equation (2), and *WS* (m/s) denotes the wind speed; RH (%) in Equation (2) is the relative humidity.

### 2.2. Statistical Analysis

The daily number of reported deaths approximately follows a Poisson distribution, because they are often regarded as rare events. Additionally, the effects of DTR and TCN were both examined non-linear on mortality [13,15]. Therefore, we built two basic DLNM models including DTR and TCN, respectively [16,17], which combined two functions that defined the exposure–response association and lag-response association to express the complicated non-linear and lagged effects. Two models were built, as follows.

DTR model:

(3) $$\log{\left(E(Y_{t1})\right)}_{DTR}=\alpha_{1}+cb(DTR_{t,l})+ns(time,df)+ns(AT,3)+\lambda_{1}Holiday_{t}+\gamma_{1}Dow_{t}$$

TCN model:

(4) $$\log{\left(E(Y_{t2})\right)}_{TCN}=\alpha_{2}+cb(TCN_{t,l})+ns(time,df)+ns(AT,3)+\lambda_{2}Holiday_{t}+\gamma_{2}Dow_{t}$$

where *t* is the observation day, *Y_t_* is the daily count of deaths, *E*(*Y_t_*) is the expected number of deaths on day *t*, *α* is the intercept and $cb\left(DTR_{t,l}\right)$ and $cb\left(TCN_{t,l}\right)$ represent the cross-basis functions obtained through the DLNM, where *l* is the maximum lag days; $cb\left(DTR_{t,l}\right)$ can be expressed as $\int_{l0}^{L}f*w\left(DTR_{t-l},l\right)dl\approx \sum_{l-l0}^{L}f*w\left(DTR_{t-l},l\right)=\sum_{l-l0}^{L}\beta_{x_{t-l},l}$. The bi-dimensional function f∗w(x,l) is defined as the exposure–lag-response function, which can model both the exposure–response curve varying with *x* and the lag-response curve varying with *l*. β represents the risk associated with the exposure, generally obtained from regression models while adjusting for potential confounders and interpreting it as the association with an exposure at the lag day versus the reference. The cross-basis function *cb*() is computed as the approximate integral of the exposure–lag-response function over the lag dimension, representing the cumulated risk over the lag period [18]. *ns*() is the natural cubic spline [19,20]; additionally, we set the maximum lag as seven days for DTR and TCN to capture the lagged effects [21,22]. The Quasi-Akaike information criterion (Q-AIC) was used to choose the optimal *df* values from the fitting models (Tables S1 and S2) [17,19]. We selected seven degrees of freedom (*df*) per year of natural cubic spline to describe the long-term trend effects both in DTR and TCN models, and for analyzing the seasonal effects, as it only needed to capture a smooth annual trend, the models were restricted to a specific season with fewer degrees of freedom (3 *df*) [23]. We also used natural cubic splines with 3 *df* for AT, as in the previous study [19,24]. *Dow_t_* (day of week) and *Holiday_t_* (public holiday) were categorical variables to control for potential confounding effects of the day of week and holiday, respectively. We chose 0 °C as the reference value for the TCN model, which meant no mean temperature change between the two adjacent days [25,26]. In addition, we selected the optimum temperature change as the reference of DTR, which corresponded to the minimum relative risk, based on the previous study [21,27,28].

After the basic models were built, we plotted the associations between DTR/TCN and mortality. To quantify the single-day and cumulative effects of DTR and TCN over different lag days, we examined the mortality risks with DTR/TCN for lag 0, 3, 0–5 and 0–7 days. Spearman’s correlation tests were applied to examine the association between meteorological factors and air pollutants to avoid collinearity, and variables with strong correlations (correlation coefficient |r| > 0.80) were not included in the same model simultaneously (Table S3).

Through subgroup analyses, we identified vulnerable populations by gender (male or female) and age (0–64 years, ≥65 years). Furthermore, we divided the full year into the cold season (October to March of the next year) and warm season (April to September) to explore the effects of seasonal variations on the association between temperature change and mortality [29,30,31].

We calculated the 95% confidence interval (CI) of the following formula to examine the differences between effect estimates at each level [32,33]:

(5) $$\left({\hat{Q}}_{1}-{\hat{Q}}_{2}\right)\pm 1.96\sqrt{{\hat{SE}}_{1}^{2}+{\hat{SE}}_{2}^{2}}$$

where ${\hat{Q}}_{1}$ and ${\hat{Q}}_{2}$ are the estimates for the two subgroups, and ${\hat{SE}}_{1}$ and ${\hat{SE}}_{2}$ are their respective standard errors.

Finally, we built a non-parametric bivariate response surface model to visualize the interaction between DTR/TCN and apparent temperature on mortality. The model was built as follows:

(6) $$\log(E(Y_{t}))=\alpha +Te(metrics,AT)+ns(time,df)+\lambda Holiday_{t}+\gamma Dow_{t}$$

where *Te*() represents a thin-plate spline, and *metrics* denotes DTR or TCN, which were the factors of interest. Other covariates were the same as the DTR and TCN models.

### 2.3. Sensitivity Analysis

To test the robustness of the model, we conducted the following sensitivity analyses: (1) changing the *df* for time of the full year (6.8 *df*) and specific season (2.4 *df*); (2) varying the *df* of apparent temperature (2.4 *df*); (3) adjusting the maximum lag days (5.14 *df*) to check the stability; (4) including air pollutants in the model; (5) adding another metrics (DTR or TCN) into the model by natural cubic spline function.

All statistics analyses were conducted with R software (version 4.0.2, R Foundation for Statistical Computing, Vienna, Austria). For all statistical tests, two-tailed *p*-values less than 0.05 were considered statistically significant.

## 3. Results

### 3.1. Descriptive Analysis

**Figure 1:**
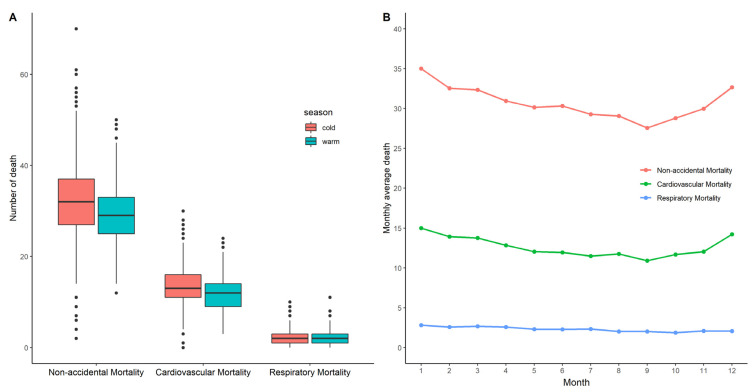
Distribution of non-accidental mortality. (**A**) Distribution of non-accidental, cardiovascular and respiratory mortality in cold and warm seasons. (**B**) Monthly average death of non-accidental, cardiovascular and respiratory mortality.

Table 1 summarizes the descriptive statistics for meteorological factors, air pollutants and daily death numbers in this study. From 2013 to 2017 in Shenzhen, the average values of DTR, TCN and AT were 5.99 °C (range: 1.30–14.00 °C), 0.004 °C (range: −10.10–6.20 °C) and 25.65 °C (range: −1.04–39.39 °C), respectively. This study included 56,034 non-accidental deaths, of which 41.4% were cardiovascular mortality and 7.5% were respiratory mortality. The proportions of males and females were 61.88% and 38.12%, whereas 54.84% were over 65 years old, and 45.16% were less than 65 years old. In addition, we recorded a daily average of 31 total non-accidental deaths, 13 cardiovascular deaths and two respiratory deaths during this study period. Finally, we found that males and the elderly (≥65 years) died more, and a larger number of deaths occurred in cold seasons (Figure 1).

### 3.2. Effects of DTR on Mortality

**Figure 2:**
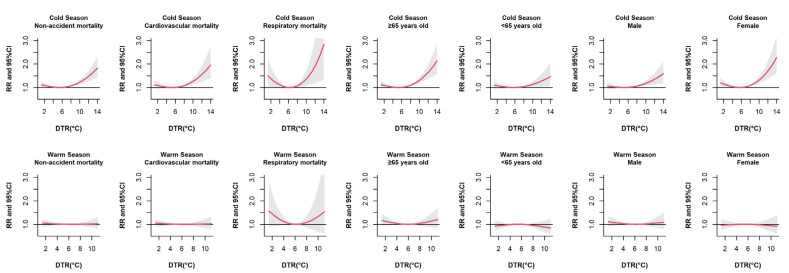
Overall exposure–response relationships between DTR and non-accidental mortality stratified by season, cause, age and sex.

**Figure 3:**
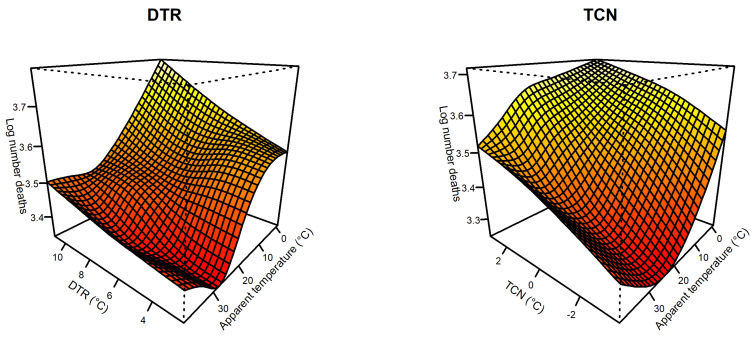
Bivariate response surface of apparent temperature and temperature change on non-accidental mortality (**Left**: lag 0–3 for DTR and lag 0 for AT; **Right**: lag 0–3 for TCN and lag 0 for AT). DTR, Diurnal temperature change; TCN, Temperature change between neighboring days.

With the point of minimum effects (5.8 °C) as the reference, the overall exposure–response relationship between DTR and non-accidental mortality followed a U-shaped but was not statistically significant (Figure S1). Figure 2 presents the relationship between DTR and non-accidental mortality by season, cause, age and sex. In cold seasons, harmful effects of high DTR were found in all subgroups. Compared to men, high DTR obviously had a greater effect on women, but both were statistically significant. In warm seasons, we did not observe significant effects between DTR and non-accidental mortality in any subgroups. Figure 3 presents the joint effects of DTR and apparent temperature on non-accidental mortality at lag 0–3. The interaction of AT and DTR showed a slight fluctuating trend, and higher DTR may increase the risk of non-accidental mortality. The lower apparent temperature increased the adverse effects of DTR, but an increase of AT would enhance the effect of DTR on non-accidental mortality when AT was higher than 28 °C, which suggests that DTR and apparent temperature have an interactive effect on non-accidental mortality.

Table 2 shows the single-day (lag 0, lag 3) and cumulative (lag 0–5, lag 0–7) lag effects of extreme high DTR (99th) for the full year and specific season. In the full year and warm seasons analyses, there were no statistically significant effects between extreme high DTR (99th) and all subgroups, except for the non-accidental and cardiovascular mortality subgroup in the full year at lag 0. However, in cold seasons, the exposure to extreme high DTR added a risk of non-accidental mortality for all subgroups, including males, females, the elderly (≥65 years) and people <65 years (Table 3).

### 3.3. Effects of TCN on Mortality

Figure S1 demonstrates the overall exposure–response relationship between TCN and mortality, which is a nearly monotonic curve with a protective effect of temperature decrease and a risky effect of temperature increase. Therefore, we focused on the effect of a large temperature change between neighboring days (99th) on mortality (Table 4). The joint association between apparent temperature and TCN at lag 0–3 is shown in Figure 3. We found roughly the same trend as that of DTR, with low apparent temperature enhancing the effect of non-accidental mortality. However, the effects of TCN increased with increasing AT when AT was higher than 25 °C. Similar interaction effects of AT and temperature change at lag 0–5 on non-accidental mortality are shown in Figure S2.

**Figure 4:**
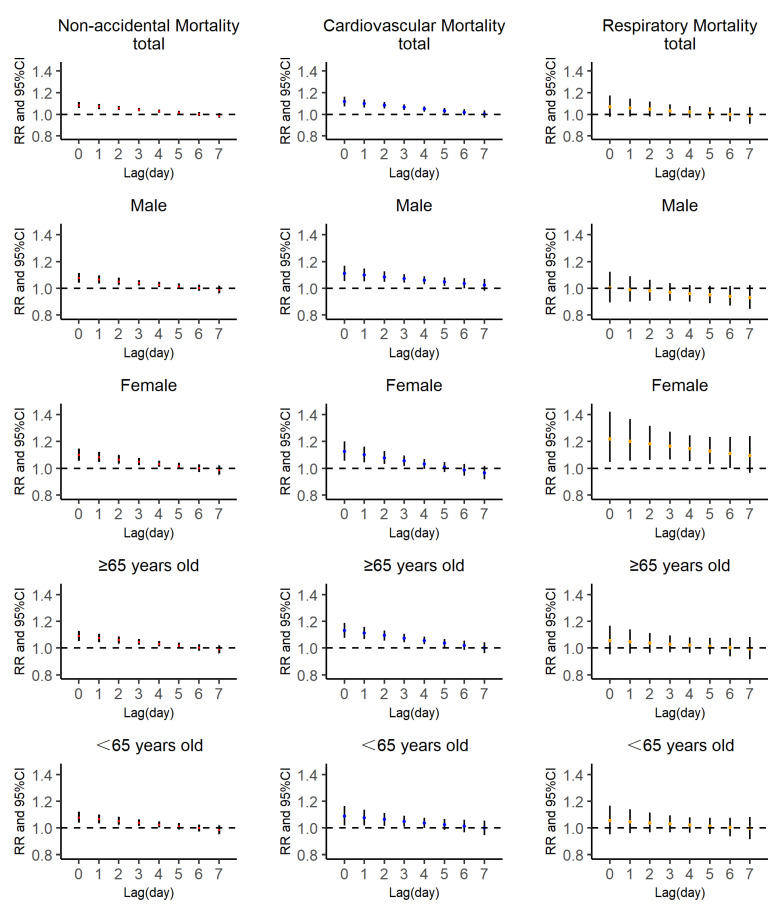
Lag-specific relative risk (RR) of non-accidental mortality in total cases and different populations with extreme high TCN (99th).

The lagged effects of extreme high TCN on mortality, stratified by cause, gender and age, are presented in Figure 4. As for the single-day lagged effect, we observed the largest effect at lag 0 (RR: 1.086, 95% CI: 1.058–1.115), then the effect decreased but remained statistically significant until lag 5 (RR: 1.016, 95% CI: 1.000–1.032). In the full year and seasonal analyses, we found that the effects of extreme high TCN were significant on non-accidental and cardiovascular mortality for all seasons. However, we only observed the significant effects of respiratory mortality in cold seasons.

### 3.4. Sensitivity Analysis

The sensitivity analyses are illustrated in Supplementary Materials (Tables S4–S12). We changed *df* for long term trends, seasonality and apparent temperature to check whether the tendency varied, and the results were stable, which indicated the robustness of our models. After adjusting the air pollutants in the model, only slight variations were observed in the results. When lag days (5, 14 days) changed, the effects exhibited a slight change. Finally, when we used the natural cubic spline function to control another temperature change metric in the model, the results changed slightly without statistically significance.

## 4. Discussion

We investigated the effects of temperature change on non-accidental mortality in Shenzhen from 2013 to 2017 using DLNMs from a time-series perspective. In this study, we used two temperature change metrics for evaluation: DTR and TCN. Temperature changes are usually impacted by many climatic effects, such as increases in cloud cover, precipitation, greenhouse gases and tropospheric aerosols, as well as local effects such as urban growth, irrigation, desertification and local land use changes [34]. Due to the local effects and the complex trends, further research on the association between temperature change and health is needed. This study indicates that large and low DTR has adverse effects on non-accidental mortality, although not statistically significant. As for TCN, we found that a positive TCN had durative adverse effects, and a negative TCN offered lasting protection; both effects were statistically significant. In addition, the female and elderly subgroups were more sensitive to large temperature changes. After adjusting the freedom of ambient temperature and air pollutants, the association did not change.

With regard to the relationship between DTR and non-accidental mortality in cold seasons, the cumulative RR was 1.407 (95% CI: 1.233–1.606) at lag 0–7 days for extreme large DTR, and we found a persistent adverse effect, which was consistent with prior studies implemented in the regions of Yuxi [35,36], Guangzhou [37] and in eight Chinese cities [12]. However, the results were inconsistent with the studies conducted in England [11]. The discrepancies could be explained as a result of the natural environment, social economy or ethnic subtypes. As for warm seasons, we observed the same curves as for cold seasons, although these were not statistically significant. Previous studies showed that DTR is significantly associated with cardiovascular and respiratory disease, such as blood pressure [6], asthma [27] and tuberculosis [38]. The exact underlying mechanism remains unclear, but there are some relevant biological explanations, such as DTR may influence humoral and cellular immune functions, increase blood pressure and induce cardiovascular disease [39]. A previous study implied that a larger DTR indicated a higher heart rate which reduced the time-domain heart rate variability after controlling for individual characteristics and environmental variables, suggesting that DTR may alter autonomic nervous functions [40]. Another explanation is that mast cells may release inflammatory mediators due to sudden ambient temperature changes and further induce some respiratory symptoms, such as cough and chest congestion [41].

In the analysis of relationships between TCN and non-accidental mortality, we found lasting significant effects in all subgroups under the extreme large exposure of TCN, and the same effects were investigated in cardiovascular mortality, whereas no such effects were found in respiratory mortality, expect for the female subgroup, in line with previous studies [13,26]. The regulation system of heat exchanges between the human body and ambient temperature has difficulty adapting when exposed to large temperature increases, which may induce the increase in blood pressure and heart rate and reduce the immune system’s resistance [42,43]. These factors are directly related to cardiovascular functions, which may be a reason why the effects of large temperature changes on respiratory mortality are not significant. Currently, we still do not know the exact mechanism of the association between negative TCN and reduced mortality; therefore, further evidence of the mechanism of action of TCN on human health is needed. In seasonal analysis, cold seasons exhibited significant effects on non-accidental mortality, cardiovascular mortality and respiratory mortality through all study periods, whereas no significant effect on respiratory mortality was found in the full year and warm seasons.

In subgroup analyses, we found discrepancies in exposure–response relationships in characteristic-specific groups (gender and age). For DTR, the elderly and females were at higher risk, although there was no difference in the comparison between groups, a fact which is supported by previous studies [22,44]. Those at increased risk may be characterized by higher exposure or intrinsic susceptibility. The adverse effects of extreme large DTR were higher for the elderly, for underlying diseases, reductions in the ability to regulate body temperature and elevation of the sweating thresholds [45,46]. Compared to females, the specific physiology of males enables better resistance to the ambient environment, and females are more sensitive to temperature change. Moreover, we found that the association between DTR and respiratory mortality was not significant in most subgroups, although the general trend was similar to non-accidental and cardiovascular mortality. It should be noted that only 7.5% of non-accidental deaths were due to respiratory mortality, making it difficult to identify the connection. Regarding TCN, lasting notable effects remained until lag 5 in all subgroups when exposed to a large TCN. However, the significant effects between TCN and respiratory mortality was only found in the female subgroup and not in any other subgroups.

Previous studies have reported that season is an important factor in modifying the association between temperature change and human health [12,13]. We explored the joint effects of temperature change and season by comparing the cause-specific mortality risk in cold and warm seasons. The cumulative effects of TCN with non-accidental mortality were 1.611 (95% CI: 1.384–1.876) in cold seasons and 1.242 (95% CI: 1.043–1.480) in warm seasons, indicating that the effects were stronger in cold seasons. Similar effects were observed for cardiovascular mortality but only found for respiratory mortality in cold seasons. In summary, cold seasons are more strongly associated with large temperature changes in comparison of warm seasons. To date, the great variations in the DTR–mortality relationship due to seasonal modification are obscure and hard to explain, and further studies are needed to investigate the underlying reasons. In this study, we did not stratify the temperature and only explored the interaction between AT and temperature change through a bivariate response surface model. The results indicated that low and high AT could notably increase temperature change effects compared to moderate temperature. At a low temperature, the risk of non-accidental mortality due to increased DTR is greater, which suggests that temperature change has an independent effect on mortality, and high DTR enhanced cold-related mortality. This is not entirely consistent with the modifying effect of temperature on DTR-mortality reported previously, as humidity or regional factors were not taken into account [47,48]. Several plausible explanations may support the potential interactive effect between temperature change and apparent temperature. Sudden temperature changes and extreme temperature have similar biological mechanisms in disrupting normal physiological thermoregulation, such as a physiological increase in blood pressure or heart rate after exposure to low temperature and rising plasma triglycerides due to large intakes of high-fat food and less exercise [49,50]. Additionally, fasting plasma glucose is higher in cold temperatures, which may be related to cardiovascular mortality [51].

The IPCC Assessment Report identified that winter would be colder and summer would be warmer due to the acceleration of climate change [52]. The government urgently needs to take effective measures against extreme weather with a large temperature change in order to reduce the corresponding economic burden. Although Shenzhen has a subtropical climate with long summers and short winters, in recent years, under the influence of tropical cyclones and monsoons, catastrophic weather events such as heavy rainfall and low-temperature frosts often occur. In addition, heavy rainfall in summer may cause a decline in apparent temperature, especially for elderly people with underlying illnesses. The government should develop and implement long-term public health management strategies and increase public health investments. In cases of extreme weather conditions, such as heavy rainfall and cold waves, timely warnings should be issued to advise the public to remain indoors more and keep warm. Additionally, hospitals should allocate and prepare medical resources to accommodate possible patient peaks. Community health centers should publicize the adverse effects of climate change and help the public to be more aware of temperature changes and prevent adverse health outcomes.

Our study had some strengths. Firstly, we chose two metrics that responded to temperature change, whose effects on non-accidental mortality have been presented comprehensively. Furthermore, we controlled for the confounding effects of meteorological factors and air pollutants; a composite index, AT, was used for modeling, and the results were convincing and objective. Finally, we explored the interactive effects between temperature change and apparent temperature, which has rarely been addressed in previous studies. The results provide a theoretical basis for governments to develop early warning programs, especially for large temperature changes with a low apparent temperature, which have a greater public health significance. Several limitations should also be mentioned. On the one hand, limited by the data availability, meteorological data were obtained from fixed monitoring sites to represent individual exposure. Moreover, the misclassification and complexity of mortality causes is inevitable. These errors may have influences on the explanation of epidemiologic studies on temperature change. On the other hand, our study did not control for other individual-level confounders, such as economic conditions, education level, occupation, etc. Air conditioning usage and time spent outdoors may have an effect on temperature change–mortality. Future research should focus on individual-level confounders and explore the specific mechanisms of temperature change on human health.

## 5. Conclusions

In summary, we found a significant association between exposure to temperature changes and non-accidental mortality in Shenzhen. The effects on cardiovascular mortality were non-negligible, whereas on respiratory mortality, they were not significant. In addition, large temperature changes may increase the risk of mortality, especially in females and elderly subgroups. Seasonal variations may alter the effects of temperature change, which cannot be ignored, and low apparent temperature enhances the effect of temperature change. Our findings have notable implications for helping governments to establish effective public health interventions and comprehensive warning systems that consider both apparent temperature and temperature change.

## Figures and Tables

**Table 1:** Summary descriptive statistics of meteorological factors, air pollutants and daily non-accidental deaths in Shenzhen, 2013–2017.

Variables	Mean (Standard Deviation)	Minimum Value	25th Percentile	50th Percentile	75th Percentile	99th Percentile	Maximum Value
Weather							
DTR (°C)	5.99 (1.98)	1.30	4.60	6.00	7.30	10.80	14.00
TCN (°C)	0.00 (1.64)	−10.10	−0.70	0.20	1.00	3.28	6.20
Apparent temperature (°C)	25.65 (8.15)	−1.04	19.19	27.25	32.97	36.55	39.39
Mean temperature (°C)	23.45 (5.49)	3.50	19.20	24.80	28.20	30.90	33.00
Relative humidity (%)	75.20 (13.16)	19.00	69.00	77.00	84.00	97.00	100.00
Wind speed (m/s)	1.97 (0.77)	0.30	1.40	1.80	2.40	4.40	5.90
Daily air pollutants							
PM_10_ (μg/m^3^)	50.80 (27.77)	8.69	29.82	43.81	65.45	138.90	181.76
NO_2_ (μg/m^3^)	39.69 (16.51)	12.00	28.32	36.10	46.97	96.55	133.71
CO (mg/m^3^)	0.95 (0.27)	0.49	0.75	0.90	1.12	1.63	1.93
SO_2_ (μg/m^3^)	9.50 (4.11)	3.52	7.00	8.55	10.80	24.94	54.81
PM_2.5_ (μg/m^3^)	32.03 (19.65)	5.64	16.76	27.72	42.28	101.57	137.07
No. of non-accidental deaths *							
Total (A00–A99)	31 (7)	2	26	30	35	49	70
Age							
Young people (<65 years)	14 (4)	1	11	14	17	25	30
Elderly (≥65 years)	17 (5)	1	14	16	20	30	40
Sex							
Male	19 (5)	1	16	19	22	32	45
Female	12 (4)	1	9	11	14	22	28
Cardiovascular disease (I00–I99)	13 (4)	0	10	12	15	24	30
Age							
Young people (<65 years)	5 (2)	0	3	4	6	11	15
Elderly (≥65 years)	8 (3)	0	6	8	10	17	20
Sex							
Male	8 (3)	0	6	8	10	16	20
Female	5 (2)	0	3	4	6	12	15
Respiratory disease (J00–J99)	2 (2)	0	1	2	3	7	11
Age							
Young people (<65 years)	0 (1)	0	0	0	1	3	5
Elderly (≥65 years)	2 (1)	0	1	2	3	6	10
Sex							
Male	2 (1)	0	1	1	2	5	9
Female	1 (1)	0	0	1	1	3	6

* Total = non-accidental deaths from all causes. DTR, Diurnal temperature change; TCN, Temperature change between neighboring days.

**Table 2:** Single-day and cumulative effects of extreme high DTR (99th) on daily non-accidental mortality for lag periods by cause and season.

Group	Lag 0	Lag 3	Lag 0–5	Lag 0–7
Non-accidental mortality				
Full year	**1.032 (1.008–1.056)**	1.010 (0.997–1.024)	1.086 (0.999–1.180)	1.055 (0.951–1.169)
Cold season	**1.063 (1.027–1.100)**	**1.046 (1.029–1.064)**	**1.333 (1.192–1.491)**	**1.407 (1.233–1.606)**
Warm season	1.014 (0.991–1.038)	1.003 (0.991–1.015)	1.028 (0.952–1.110)	1.007 (0.920–1.101)
Cardiovascular mortality				
Full year	**1.037 (1.002–1.074)**	1.018 (0.998–1.038)	1.133 (0.999–1.286)	1.124 (0.961–1.314)
Cold season	**1.066 (1.015–1.119)**	**1.052 (1.027–1.077)**	**1.371 (1.171–1.606)**	**1.470 (1.220–1.771)**
Warm season	1.031 (0.984–1.080)	1.006 (0.983–1.029)	1.063 (0.914–1.236)	1.016 (0.851–1.213)
Respiratory mortality				
Full year	0.961 (0.891–1.036)	0.986 (0.948–1.025)	0.895 (0.696–1.151)	0.924 (0.683–1.250)
Cold season	1.077 (0.969–1.198)	**1.073 (1.017–1.131)**	**1.529 (1.082–2.162)**	**1.741 (1.157–2.620)**
Warm season	0.999 (0.901–1.108)	1.016 (0.967–1.069)	1.084 (0.778–1.511)	1.165 (0.796–1.706)

Bold values represent statistically significant results (*p* < 0.05). The 99th percentile of DTR distribution (10.8 °C for the full year, 11.5 °C for the cold season and 8.8 °C for the warm season), with reference to the minimum relative risk in each subgroup.

**Table 3:** Relative risks (95% CI) of daily non-accidental mortality associated with extreme high DTR (99th) by age and sex in the specific season.

Group	Lag 0	Lag 3	Lag 0–5	Lag 0–7
Full year				
Male	1.027 (0.998–1.057)	1.008 (0.992–1.025)	1.070 (0.964–1.188)	1.043 (0.917–1.187)
Female	**1.038 (1.002–1.076)**	1.013 (0.992–1.034)	1.107 (0.973–1.260)	1.072 (0.913–1.259)
≥65 years old	1.016 (0.986–1.047) *	1.016 (0.999–1.034)	1.102 (0.989–1.229)	1.140 (0.997–1.303)
<65 years old	**1.044 (1.011–1.077)**	0.999 (0.982–1.015)	1.036 (0.933–1.152)	0.932 (0.821–1.058)
Cold season				
Male	**1.051 (1.009–1.095)**	**1.036 (1.016–1.057)**	1.256 (1.100–1.434) *	1.306 (1.118–1.527) *
Female	**1.082 (1.031–1.137)**	**1.063 (1.037–1.089)**	**1.467 (1.250–1.721)**	**1.587 (1.314–1.917)**
≥65 years old	1.030 (0.987–1.075) *	**1.052 (1.031–1.075)**	**1.330 (1.158–1.527)**	1.548 (1.317–1.820) *
<65 years old	**1.107 (1.056–1.160)**	**1.038 (1.014–1.062)**	**1.333 (1.144–1.553)**	**1.235 (1.028–1.482)**
Warm season				
Male	1.018 (0.983–1.054)	1.006 (0.989–1.023)	1.048 (0.935–1.174)	1.031 (0.903–1.177)
Female	1.016 (0.968–1.066)	0.998 (0.975–1.022)	1.007 (0.860–1.178)	0.963 (0.802–1.157)
≥65 years old	1.039 (0.999–1.080)	1.013 (0.994–1.033)	1.108 (0.977–1.257)	1.073 (0.926–1.243)
<65 years old	0.992 (0.950–1.036)	0.991 (0.970–1.012)	0.948 (0.824–1.091)	0.928 (0.788–1.093)

Bold values represent statistically significant results (*p* < 0.05). * Significant results of Z tests for the difference between the two relative risks of subgroup analysis (*p* < 0.05). The 99th percentile of DTR distribution (10.8 °C for the full year, 11.5 °C for the cold season and 8.8 °C for the warm season), with reference to the minimum relative risk in each subgroup.

**Table 4:** Single-day and cumulative effects of extreme high TCN (99th) on daily non-accidental mortality for lag periods by cause and season.

Group	Lag 0	Lag 3	Lag 0–5	Lag 0–7
Non-accidental mortality				
Full year	**1.086 (1.058–1.115)**	**1.043 (1.027–1.060)**	**1.342 (1.213–1.486)**	**1.331 (1.180–1.500)**
Cold season	**1.132 (1.091–1.175)**	**1.071 (1.050–1.093)**	**1.258 (1.177–1.345)**	**1.611 (1.384–1.876)**
Warm season	**1.063 (1.023–1.106)**	**1.033 (1.009–1.056)**	**1.248 (1.077–1.445)**	**1.242 (1.043–1.480)**
Cardiovascular mortality				
Full year	**1.117 (1.073–1.163)**	**1.066 (1.041–1.092)**	**1.538 (1.317–1.795)**	**1.567 (1.304–1.881)**
Cold season	**1.145 (1.087–1.207)**	**1.087 (1.056–1.118)**	**1.289 (1.172–1.417)**	**1.814 (1.462–2.250)**
Warm season	**1.112 (1.045–1.183)**	**1.052 (1.015–1.091)**	**1.434 (1.136–1.811)**	**1.398 (1.058–1.847)**
Respiratory mortality				
Full year	1.071 (0.976–1.175)	1.034 (0.978–1.093)	1.265 (0.885–1.809)	1.246 (0.817–1.899)
Cold season	**1.178 (1.048–1.324)**	**1.089 (1.022–1.160)**	**1.352 (1.095–1.669)**	**1.779 (1.096–2.887)**
Warm season	1.081 (0.933–1.253)	1.085 (0.996–1.183)	1.627 (0.933–2.838)	1.935 (0.999–3.746)

Bold values represent statistically significant results (*p* < 0.05). The 99th percentile of TCN distribution (3.3 °C for the full year, 3.9 °C for the cold season and 2.7 °C for the warm season) with a reference of 0 °C.

## Data Availability

The data are not publicly available due to the agreement of privacy protection with the data provider.

## Supplementary Materials

The following are available online at https://www.mdpi.com/article/10.3390/ijerph18168760/s1, Table S1: Quasi-Akaike information criterion (Q-AIC) values for DTR-model, Table S2: Quasi-Akaike information criterion (Q-AIC) values for TCN-model, Table S3: Spearman’s correlation coefficients among different air pollutant concentrations and meteorological factors in Shenzhen from 2013 to 2017, Table S4 Relative risk (95% CI) of non-accidental mortality for extreme high DTR at single-day and cumulative lag periods by varying modeling choices, Table S5: Relative risk (95% CI) of non-accidental mortality for extreme high DTR at single-day and cumulative lag periods with adjustment for the TCN variable, Table S6: Relative risk (95% CI) of non-accidental mortality for extreme high DTR at single-day and cumulative lag periods with adjustment for co-pollutants, Table S7: Relative risk (95% CI) of non-accidental mortality for extreme high DTR at single-day and cumulative lag periods with adjustment for apparent temperature, Table S8: Relative risk (95% CI) of non-accidental mortality for extreme high DTR at single-day and cumulative lag periods with adjustment for lag periods, Table S9: Relative risk (95% CI) of non-accidental mortality for extreme high TCN at single-day and cumulative lag periods by varying modeling choices, Table S10: Relative risk (95% CI) of non-accidental mortality for extreme high TCN at single-day and cumulative lag periods with adjustment for the DTR variable, Table S11: Relative risk (95% CI) of non-accidental mortality for extreme high TCN at single-day and cumulative lag periods with adjustment for co-pollutants, Table S12: Relative risk (95% CI) of non-accidental mortality for extreme high TCN at single-day and cumulative lag periods with adjustment for apparent temperature, Figure S1: Exposure–response relationship between temperature change and risk of non-accidental mortality. Left panels: 3D plots of the exposure–lag-response risks. Right panels: overall exposure–response associations with diurnal temperature change (DTR) and temperature change between neighboring days (TCN), Figure S2: Bivariate response surface of apparent temperature and temperature change on non-accidental mortality (Left: lag 0–5 for DTR and lag 0 for AT; right: lag 0–5 for TCN and lag 0 for AT).

## References

[1] IPCC (2013). Climate Change 2014 Synthesis Report Summary for Policymakers. https://www.ipcc.ch/report/ar5/syr/.

[2] Yang Z., Wang Q., Liu P. (2018). Extreme temperature and mortality: Evidence from China. Int. J. Biometeorol..

[3] Cheng J., Xu Z., Bambrick H., Su H., Tong S., Hu W. (2018). Impacts of heat, cold, and temperature variability on mortality in Australia, 2000–2009. Sci. Total Environ..

[4] Zha Q., Chai G., Zhang Z.-G., Sha Y., Su Y. (2021). Effects of diurnal temperature range on cardiovascular disease hospital admissions in farmers in China’s Western suburbs. Environ. Sci. Pollut. Res..

[5] Yang J., Zhou M., Li M., Yin P., Wang B., Pilot E., Liu Y., van der Hoek W., van Asten L., Krafft T. (2018). Diurnal temperature range in relation to death from stroke in China. Environ. Res..

[6] Zheng S., Zhu W., Wang M., Shi Q., Luo Y., Miao Q., Nie Y., Kang F., Mi X., Bai Y. (2020). The effect of diurnal temperature range on blood pressure among 46,609 people in Northwestern China. Sci. Total Environ..

[7] Gostimirovic M., Novakovic R., Rajkovic J., Djokic V., Terzic D., Putnik S., Gojkovic-Bukarica L. (2020). The influence of climate change on human cardiovascular function. Arch. Environ. Occup. Health.

[8] Zhang B., Li G., Ma Y., Pan X. (2018). Projection of temperature-related mortality due to cardiovascular disease in beijing under different climate change, population, and adaptation scenarios. Environ. Res..

[9] Yang J., Zhou M., Li M., Liu X., Yin P., Sun Q., Wang J., Wu H., Wang B., Liu Q. (2018). Vulnerability to the impact of temperature variability on mortality in 31 major Chinese cities. Environ. Pollut..

[10] Ma C., Yang J., Nakayama S.F., Honda Y. (2019). The association between temperature variability and cause-specific mortality: Evidence from 47 Japanese prefectures during 1972–2015. Environ. Int..

[11] Zhang Y., Peng M., Wang L., Yu C. (2018). Association of diurnal temperature range with daily mortality in England and Wales: A nationwide time-series study. Sci. Total Environ..

[12] Zhou X., Zhao A., Meng X., Chen R., Kuang X., Duan X., Kan H. (2014). Acute effects of diurnal temperature range on mortality in 8 Chinese cities. Sci. Total Environ..

[13] Zhan Z., Zhao Y., Pang S., Zhong X., Wu C., Ding Z. (2017). Temperature change between neighboring days and mortality in United States: A nationwide study. Sci. Total Environ..

[14] Steadman R.G. (1993). Norms of apparent temperature in Australia. Aust. Meterol. Mag..

[15] Yang J., Liu H.-Z., Ou C.-Q., Lin G.-Z., Zhou Q., Shen G.-C., Chen P.-Y., Guo Y. (2013). Global climate change: Impact of diurnal temperature range on mortality in Guangzhou, China. Environ. Pollut..

[16] Armstrong B. (2006). Models for the Relationship Between Ambient Temperature and Daily Mortality. Epidemiology.

[17] Gasparrini A. (2014). Modeling exposure-lag-response associations with distributed lag non-linear models. Stat. Med..

[18] Gasparrini A., Leone M. (2014). Attributable risk from distributed lag models. BMC Med. Res. Methodol..

[19] Gasparrini A., Armstrong B., Kenward M.G. (2010). Distributed lag non-linear models. Stat. Med..

[20] Gasparrini A. (2011). Distributed Lag Linear and Non-Linear Models inR: The Packagedlnm. J. Stat. Softw..

[21] Huang K., Yang X.-J., Hu C.-Y., Ding K., Jiang W., Hua X.-G., Liu J., Cao J.-Y., Sun C.-Y., Zhang T. (2020). Short-term effect of ambient temperature change on the risk of tuberculosis admissions: Assessments of two exposure metrics. Environ. Res..

[22] Deng J., Hu X., Xiao C., Xu S., Gao X., Ma Y., Yang J., Wu M., Liu X., Ni J. (2019). Ambient temperature and non-accidental mortality: A time series study. Environ. Sci. Pollut. Res..

[23] Wang M.-Z., Zheng S., He S.-L., Li B., Teng H.-J., Wang S.-G., Yin L., Shang K.-Z., Li T.-S. (2013). The association between diurnal temperature range and emergency room admissions for cardiovascular, respiratory, digestive and genitourinary disease among the elderly: A time series study. Sci. Total Environ..

[24] Zhao Y., Huang Z., Wang S., Hu J., Xiao J., Li X., Liu T., Zeng W., Guo L., Du Q. (2019). Morbidity burden of respiratory diseases attributable to ambient temperature: A case study in a subtropical city in China. Environ. Health.

[25] Cheng J., Zhu R., Xu Z., Xu X., Wang X., Li K., Su H. (2014). Temperature variation between neighboring days and mortality: A distributed lag non-linear analysis. Int. J. Public Health.

[26] Lin H., Zhang Y., Xu Y., Xu X., Liu T., Luo Y., Xiao J., Wu W., Ma W. (2013). Temperature Changes between Neighboring Days and Mortality in Summer: A Distributed Lag Non-Linear Time Series Analysis. PLoS ONE.

[27] Wei Q., Zhong L., Gao J., Yi W., Pan R., Gao J., Duan J., Xu Z., He Y., Liu X. (2020). Diurnal temperature range and childhood asthma in Hefei, China: Does temperature modify the association?. Sci. Total Environ..

[28] Gasparrini A., Guo Y., Hashizume M., Lavigne E., Zanobetti A., Schwartz J., Tobías A., Tong S., Rocklöv J., Forsberg B. (2015). Mortality risk attributable to high and low ambient temperature: A multicountry observational study. Lancet.

[29] Cao J., Cheng Y., Zhao N., Song W., Jiang C., Chen R., Kan H. (2009). Diurnal Temperature Range is a Risk Factor for Coronary Heart Disease Death. J. Epidemiol..

[30] Chit-Ming Wong S.M., Hedley A.J., Lam T.-H. (2001). Effect of Air Pollution on Daily Mortality in Hong Kong. Environ. Health Perspect..

[31] Liu T., Li T.T., Zhang Y.H., Xu Y.J., Lao X.Q., Rutherford S., Chu C., Luo Y., Zhu Q., Xu X.J. (2012). The short-term effect of ambient ozone on mortality is modified by temperature in Guangzhou, China. Atmos. Environ..

[32] Zeka A., Zanobetti A., Schwartz J. (2006). Individual-Level Modifiers of the Effects of Particulate Matter on Daily Mortality. Am. J. Epidemiol..

[33] Schenker N., Gentleman J.F. (2001). On Judging the Significance of Differences by Examining the Overlap Between Confidence Intervals. Am. Stat..

[34] Kan H., London S., Chen H., Song G., Chen G., Jiang L., Zhao N., Zhang Y., Chen B. (2007). Diurnal temperature range and daily mortality in Shanghai, China. Environ. Res..

[35] Ding Z., Guo P., Xie F., Chu H., Li K., Pu J., Pang S., Dong H., Liu Y., Pi F. (2015). Impact of diurnal temperature range on mortality in a high plateau area in southwest China: A time series analysis. Sci. Total Environ..

[36] Ding Z., Li L., Xin L., Pi F., Dong W., Wen Y., Au W.W., Zhang Q. (2016). High diurnal temperature range and mortality: Effect modification by individual characteristics and mortality causes in a case-only analysis. Sci. Total Environ..

[37] Luo Y., Zhang Y., Liu T., Rutherford S., Xu Y., Xu X., Wu W., Xiao J., Zeng W., Chu C. (2013). Lagged Effect of Diurnal Temperature Range on Mortality in a Subtropical Megacity of China. PLoS ONE.

[38] Wang B., Chai G., Sha Y., Zha Q., Su Y., Gao Y. (2021). Impact of ambient temperature on cardiovascular disease hospital admissions in farmers in China’s Western suburbs. Sci. Total Environ..

[39] Bull G. (1980). The weather and deaths from pneumonia. Lancet.

[40] Tang M., He Y., Zhang X., Li H., Huang C., Wang C., Gao Y., Li Y., Kan H., Hu J. (2020). The acute effects of temperature variability on heart rate variability: A repeated-measure study. Environ. Res..

[41] Togias A.G., Naclerio R.M., Proud D., Fish J.E., Adkinson N.F., Kagey-Sobotka A., Norman P.S., Lichtenstein L.M. (1985). Nasal challenge with cold, dry air results in release of inflammatory mediators. Possible mast cell involvement. J. Clin. Investig..

[42] Zanobetti A., O’Neill M.S., Gronlund C.J., Schwartz J.D. (2012). Summer temperature variability and long-term survival among elderly people with chronic disease. Proc. Natl. Acad. Sci. USA.

[43] Guo Y., Barnett A., Yu W., Pan X., Ye X., Huang C., Tong S. (2011). A Large Change in Temperature between Neighbouring Days Increases the Risk of Mortality. PLoS ONE.

[44] Ma Y., Zhao Y., Zhou J., Jiang Y., Yang S., Yu Z. (2018). The relationship between diurnal temperature range and COPD hospital admissions in Changchun, China. Environ. Sci. Pollut. Res..

[45] Kenney W.L., Hodgson I.L. (1987). Heat tolerance, thermoregulation and ageing. Sports Med..

[46] Lee J.-B., Kim J.-H., Murota H. (2016). Perspiration Functions in Different Ethnic, Age, and Sex Populations: Modification of Sudomotor Function. Perspiration Res..

[47] Lee W., Chung Y., Choi H.M., Kim D., Honda Y., Guo Y.L., Kim H. (2019). Interactive Effect of Diurnal Temperature Range and Temperature on Mortality, Northeast Asia. Epidemiology.

[48] Lee W., Kim Y., Honda Y., Kim H. (2018). Association between diurnal temperature range and mortality modified by temperature in Japan, 1972–2015: Investigation of spatial and temporal patterns for 12 cause-specific deaths. Environ. Int..

[49] Madaniyazi L., Zhou Y., Li S., Williams G., Jaakkola J., Liang X., Liu Y., Wu S., Guo Y. (2016). Outdoor Temperature, Heart Rate and Blood Pressure in Chinese Adults: Effect Modification by Individual Characteristics. Sci. Rep..

[50] Zhou X., Lin H., Zhang S., Ren J., Wang Z., Zhang Y., Wang M., Zhang Q. (2016). Effects of climatic factors on plasma lipid levels: A 5-year longitudinal study in a large Chinese population. J. Clin. Lipidol..

[51] Li S., Zhou Y., Williams G., Jaakkola J., Ou C., Chen S., Yao T., Qin T., Wu S., Guo Y. (2016). Seasonality and temperature effects on fasting plasma glucose: A population-based longitudinal study in China. Diabetes Metab..

[52] Allen S., Cardona O., Cutter S., Dube O.P., Ebi K., Handmer J., Lavell A., Mastrandrea M., McBean G., Mechler R. (2012). Managing the Risks of Extreme Events and Disasters to Advance Climate Change Adaptation. Special Report of Working Groups I and II of the Intergovernmental Panel on Climate Change.

