# Supplementary material for: Short-Term Effect of Temperature Change on Non-Accidental Mortality in Shenzhen, China
Source: Int J Environ Res Public Health. 2021 Aug 19;18(16):8760. doi: 10.3390/ijerph18168760 (PMC8392083; doi:10.3390/ijerph18168760)
Supplement: Supplementary file 1 [file ijerph-18-08760-s001.zip › ijerph-1303458-supplementary.pdf]

Supplementary material

Short-Term Effect of Temperature Change on Non-Accidental Mortality in Shenzhen, China

Yao Xiao, Chengzhen Meng, Suli Huang, Yanran Duan, Gang Liu, Shuyuan Yu,
Ji Peng, Jinquan Cheng, Ping Yin

List of Tables and Figures

Table S1 Quasi-Akaike information criterion (Q-AIC) values for DTR-model.

Table S2 Quasi-Akaike information criterion (Q-AIC) values for TCN-model.

Table S3 Spearman's correlation coefficients among different air pollutant concentrations and meteorological factors in Shenzhen from 2013 to 2017.

Table S4 Relative risk (95% *CI*) of non-accidental mortality for extreme high DTR at single-day and cumulative lag periods by varying modeling choices.

Table S5 Relative risk (95% *CI*) of non-accidental mortality for extreme high DTR at single-day and cumulative lag periods with adjustment for the TCN variable.

Table S6 Relative risk (95% *CI*) of non-accidental mortality for extreme high DTR at single-day and cumulative lag periods with adjustment for co-pollutants.

Table S7 Relative risk (95% *CI*) of non-accidental mortality for extreme high DTR at single-day and cumulative lag periods with adjustment for apparent temperature.

Table S8 Relative risk (95% *CI*) of non-accidental mortality for extreme high DTR at single-day and cumulative lag periods with adjustment for lag periods.

Table S9 Relative risk (95% *CI*) of non-accidental mortality for extreme high TCN at single-day and cumulative lag periods by varying modeling choices.

Table S10 Relative risk (95% *CI*) of non-accidental mortality for extreme high TCN at single-day and cumulative lag periods with adjustment for the DTR variable.

Table S11 Relative risk (95% *CI*) of non-accidental mortality for extreme high TCN at single-day and cumulative lag periods with adjustment for co-pollutants.

Table S12 Relative risk (95% *CI*) of non-accidental mortality for extreme high TCN at single-day and cumulative lag periods with adjustment for apparent temperature.

Figure S1. Exposure–response relationship between temperature change and risk of non-accidental mortality. Left panels: 3D plots of the exposure–lag-response risks. Right panels: overall exposure–response associations with diurnal temperature change (DTR) and temperature change between neighboring days (TCN).

Figure S2. Bivariate response surface of apparent temperature and temperature change on non-accidental mortality (Left: lag 0–5 for DTR and lag 0 for AT; right: lag 0–5 for TCN and lag 0 for AT).

Table S1. Quasi-Akaike information criterion (Q-AIC) [a] values for DTR-model.

df for DTR	df for DTR lag				
	**2**	**3**	**4**	**5**	**6**
2	**11664.12**	11668.21	11669.57	11671.65	11670.98
3	11668.56	11674.90	11677.82	11681.42	11683.41
4	11664.46	11672.75	11676.73	11682.40	11685.56
5	11668.38	11677.94	11684.11	11688.37	11694.21
6	11671.60	11683.56	11690.40	11696.14	11703.76

[a] Quasi Akaike Information Criterion, Q-AIC; its corresponding formula is as follows: Q-AIC = −2(maximum log-likelihood) + 2df* $\hat{\varphi}$, where $\hat{\varphi}$ is the estimated overdispersion parameter.

Table S2. Quasi-Akaike information criterion (Q-AIC) [a] values for TCN-model.

df for TCN	df for TCN lag				
	**2**	**3**	**4**	**5**	**6**
2	**11617.30**	11622.66	11626.89	11631.45	11633.25
3	11620.33	11625.39	11631.84	11638.71	11642.95
4	11623.62	11631.03	11639.00	11647.64	11654.21
5	11626.04	11627.95	11635.78	11646.73	11655.07
6	11618.19	11619.53	11629.69	11642.78	11652.39

[a] Quasi Akaike Information Criterion, Q-AIC; its corresponding formula is as follows: Q-AIC = −2(maximum log-likelihood) + 2df* $\hat{\varphi}$, where $\hat{\varphi}$ is the estimated overdispersion parameter

Table S3. Spearman's correlation coefficients among different air pollutant concentrations and meteorological factors in Shenzhen from 2013 to 2017.

	Apparent temperature	DTR	TCN	CO	NO_2	SO_2	PM_{10}	$PM_{2.5}$	O_3
Apparent temperature	1.00	−0.09*	0.07*	−0.44*	−0.34*	−0.14*	−0.48*	−0.56*	−0.07*
DTR		1.00	0.33*	0.11*	0.27*	0.42*	0.36*	0.31*	0.21*
TCN			1.00	−0.01	0.22*	0.21*	0.18*	0.14*	0.01
CO				1.00	0.55*	0.30*	0.51*	0.54*	−0.17*
NO2					1.00	0.56*	0.60*	0.59*	−0.21*
SO2						1.00	0.63*	0.57*	0.06*
PM10							1.00	0.96*	0.33*
PM2.5								1.00	0.33*
O3									1.00

Abbreviations: DTR, diurnal temperature change; TCN, temperature change between neighboring days; CO, carbon monoxide; NO_2, nitrogen dioxide; SO_2, sulfur dioxide; PM_{10}, particulate matter less than 10μm in aerodynamic diameter; $PM_{2.5}$, particulate matter less than 2.5μm in aerodynamic diameter; O_3, ozone.

[*] $p < 0.01$.

Table S4. Relative risk (95% *CI*) of non-accidental mortality for extreme high DTR at single-day and cumulative lag periods by varying modeling choices.

	Modeling choice	Lag 0	Lag 3	Lag 0–5	Lag 0–7
Full year					
Non-accidental mortality	Main model	**1.032(1.008-1.056)**	1.010(0.997-1.024)	1.086(0.999-1.180)	1.055(0.951-1.169)
Cardiovascular mortality		**1.037(1.002-1.074)**	1.018(0.998-1.038)	1.133(0.999-1.286)	1.124(0.961-1.314)
Respiratory mortality		0.961(0.891-1.036)	0.986(0.948-1.025)	0.895(0.696-1.151)	0.924(0.683-1.250)
Male		1.027(0.998-1.057)	1.008(0.992-1.025)	1.070(0.964-1.188)	1.043(0.917-1.187)
Female		**1.038(1.002-1.076)**	1.013(0.992-1.034)	1.107(0.973-1.260)	1.072(0.913-1.259)
≥65 years old		1.016(0.986-1.047)*	1.016(0.999-1.034)	1.102(0.989-1.229)	1.140(0.997-1.303)
<65 years old		**1.044(1.011-1.077)**	0.999(0.982-1.015)	1.036(0.933-1.152)	0.932(0.821-1.058)
Non-accidental mortality	Df /year for time trend：6	**1.032(1.009-1.056)**	1.010(0.997-1.023)	**1.084(1.000-1.176)**	1.051(0.951-1.162)
Cardiovascular mortality		**1.038(1.003-1.075)**	1.017(0.998-1.037)	**1.130(1.000-1.278)**	1.115(0.958-1.298)
Respiratory mortality		0.993(0.922-1.069)	0.987(0.948-1.028)	0.930(0.718-1.206)	0.894(0.650-1.229)
Male		1.026(0.997-1.056)	1.007(0.991-1.023)	1.063(0.960-1.177)	1.031(0.909-1.169)
Female		**1.041(1.004-1.078)**	1.014(0.994-1.034)	1.115(0.984-1.265)	1.080(0.925-1.261)
≥65 years old		1.014(0.984-1.044)*	1.013(0.997-1.030)	1.082(0.975-1.201)	1.109(0.976-1.261)
＜65 years old		**1.050(1.017-1.084)**	1.002(0.986-1.018)	1.060(0.955-1.177)	0.954(0.842-1.082)
Non-accidental mortality	Df /year for time trend：8	**1.031(1.008-1.055)**	1.009(0.996-1.022)	1.078(0.992-1.172)	1.042(0.940-1.156)
Cardiovascular mortality		**1.038(1.002-1.075)**	1.018(0.998-1.038)	1.133(0.998-1.288)	1.122(0.958-1.314)
Respiratory mortality		0.960(0.890-1.035)	0.984(0.947-1.024)	0.887(0.689-1.143)	0.913(0.673-1.238)
Male		1.027(0.998-1.057)	1.008(0.991-1.024)	1.067(0.960-1.185)	1.036(0.909-1.180)
Female		**1.038(1.002-1.075)**	1.011(0.991-1.031)	1.095(0.966-1.242)	1.052(0.900-1.229)
≥65 years old		1.014(0.984-1.045)*	1.015(0.997-1.032)	1.090(0.978-1.215)	1.123(0.982-1.283)
＜65 years old		**1.055(1.023-1.088)**	1.001(0.984-1.019)	1.062(0.951-1.185)	0.942(0.822-1.080)
Cold season					
Non-accidental mortality	Main model	**1.063(1.027-1.100)**	**1.046(1.029-1.064)**	**1.333(1.192-1.491)**	**1.407(1.233-1.606)**
Cardiovascular mortality		**1.066(1.015-1.119)**	**1.052(1.027-1.077)**	**1.371(1.171-1.606)**	**1.470(1.220-1.771)**
Respiratory mortality		1.077(0.969-1.198)	**1.073(1.017-1.131)**	**1.529(1.082-2.162)**	**1.741(1.157-2.620)**
Male		**1.051(1.009-1.095)**	**1.036(1.016-1.057)**	**1.256(1.100-1.434)***	**1.306(1.118-1.527)***
Female		**1.082(1.031-1.137)**	**1.063(1.037-1.089)**	**1.467(1.250-1.721)**	**1.587(1.314-1.917)**

≥65 years old		1.030(0.987-1.075)*	**1.052(1.031-1.075)**	**1.330(1.158-1.527)**	**1.548(1.317-1.820)***
<65 years old		**1.107(1.056-1.160)**	**1.038(1.014-1.062)**	**1.333(1.144-1.553)**	**1.235(1.028-1.482)**
Non-accidental mortality	Df for time trend:2	**1.061(1.024-1.099)**	**1.045(1.027-1.063)**	**1.322(1.179-1.482)**	**1.394(1.218-1.595)**
Cardiovascular mortality		**1.065(1.014-1.118)**	**1.051(1.026-1.076)**	**1.363(1.164-1.598)**	**1.458(1.209-1.759)**
Respiratory mortality		1.077(0.966-1.201)	**1.071(1.014-1.131)**	**1.517(1.063-2.165)**	**1.716(1.128-2.612)**
Male		**1.049(1.006-1.093)**	**1.035(1.014-1.057)**	**1.246(1.088-1.4260***	**1.294(1.103-1.517)***
Female		**1.081(1.028-1.136)**	**1.061(1.035-1.088)**	**1.455(1.237-1.711)**	**1.570(1.296-1.902)**
≥65 years old		1.028(0.984-1.074)*	**1.051(1.029-1.074)**	**1.319(1.146-1.518)**	**1.534(1.301-1.809)***
<65 years old		**1.105(1.054-1.159)**	**1.036(1.012-1.061)**	**1.322(1.132-1.543)**	**1.222(1.015-1.471)**
Non-accidental mortality	Df for time trend:4	**1.056(1.021-1.093)**	**1.041(1.024-1.059)**	**1.294(1.157-1.448)**	**1.358(1.189-1.551)**
Cardiovascular mortality		**1.061(1.011-1.114)**	**1.047(1.022-1.073)**	**1.337(1.141-1.567)**	**1.423(1.179-1.717)**
Respiratory mortality		1.075(0.966-1.196)	**1.071(1.015-1.129)**	**1.512(1.067-2.143)**	**1.716(1.136-2.592)**
Male		**1.046(1.004-1.090)**	**1.033(1.012-1.054)**	**1.228(1.075-1.403)**	**1.270(1.086-1.486)**
Female		**1.074(1.023-1.128)**	**1.056(1.030-1.082)**	**1.411(1.201-1.657)**	**1.511(1.249-1.829)**
≥65 years old		1.024(0.981-1.069)*	**1.048(1.025-1.070)**	**1.292(1.123-1.486)**	**1.495(1.269-1.762)***
<65 years old		**1.100(1.050-1.153)**	**1.033(1.009-1.057)**	**1.293(1.108-1.507)**	1.190(0.990-1.431)
Warm season					
Non-accidental mortality	Main model	1.014(0.991-1.038)	1.003(0.991-1.015)	1.028(0.952-1.110)	1.007(0.920-1.101)
Cardiovascular mortality		1.031(0.984-1.080)	1.006(0.983-1.029)	1.063(0.914-1.236)	1.016(0.851-1.213)
Respiratory mortality		0.999(0.901-1.108)	1.016(0.967-1.069)	1.084(0.778-1.511)	1.165(0.796-1.706)
Male		1.018(0.983-1.054)	1.006(0.989-1.023)	1.048(0.935-1.174)	1.031(0.903-1.177)
Female		1.016(0.968-1.066)	0.998(0.975-1.022)	1.007(0.860-1.178)	0.963(0.802-1.157)
≥65 years old		1.039(0.999-1.080)	1.013(0.994-1.033)	1.108(0.977-1.257)	1.073(0.926-1.243)
<65 years old		0.992(0.950-1.036)	0.991(0.970-1.012)	0.948(0.824-1.091)	0.928(0.788-1.093)
Non-accidental mortality	Df for time trend:2	1.015(0.991-1.039)	1.003(0.991-1.015)	1.029(0.953-1.111)	1.008(0.921-1.102)
Cardiovascular mortality		1.030(0.985-1.078)	1.006(0.984-1.029)	1.061(0.915-1.230)	1.015(0.854-1.207)
Respiratory mortality		1.002(0.904-1.111)	1.017(0.968-1.069)	1.091(0.784-1.519)	1.170(0.801-1.711)
Male		1.018(0.983-1.054)	1.006(0.989-1.023)	1.049(0.936-1.175)	1.032(0.904-1.178)
Female		1.016(0.968-1.067)	0.998(0.975-1.022)	1.007(0.860-1.178)	0.962(0.801-1.155)
≥65 years old		1.039(0.998-1.081)	1.013(0.993-1.033)	1.109(0.974-1.261)	1.073(0.923-1.248)
<65 years old		0.994(0.952-1.038)	0.991(0.970-1.012)	0.950(0.825-1.093)	0.927(0.787-1.092)
Non-accidental mortality	Df for time	1.017(0.990-1.044)	1.004(0.991-1.017)	1.036(0.950-1.129)	1.013(0.916-1.120)

Cardiovascular mortality	trend:4	1.035(0.988-1.085)	1.010(0.986-1.033)	1.085(0.931-1.265)	1.044(0.872-1.249)
Respiratory mortality		0.998(0.899-1.107)	1.014(0.965-1.067)	1.072(0.767-1.497)	1.147(0.781-1.685)
Male		1.020(0.984-1.057)	1.007(0.990-1.026)	1.058(0.940-1.190)	1.044(0.909-1.198)
Female		1.015(0.967-1.066)	0.998(0.974-1.022)	1.004(0.857-1.176)	0.959(0.797-1.154)
⩾65 years old		**1.040(1.000-1.082)**	1.014(0.995-1.034)	1.117(0.981-1.271)	1.083(0.931-1.260)
＜65 years old		0.993(0.951-1.037)	0.991(0.970-1.013)	0.951(0.826-1.095)	0.931(0.790-1.098)

Bold values represent statistically significant results ($p < 0.05$).

* Significant results of Z tests for the difference between the two relative risks of subgroup analysis ($p < 0.05$).

The 99th percentile of DTR distribution (10.8 °C for the full year, 11.5 °C for the cold season and 8.8 °C for the warm season) with reference to the minimum relative risk in each subgroup.

Table S5. Relative risk (95% *CI*) of non-accidental mortality for extreme high DTR at single-day and cumulative lag periods with adjustment for the TCN variable.

	**Lag 0**	**Lag 3**	**Lag 0–5**	**Lag 0–7**
Full year				
Non-accidental mortality	**1.029(1.005-1.053)**	1.010(0.996-1.023)	1.079(0.992-1.174)	1.054(0.950-1.169)
Cardiovascular mortality	1.032(0.996-1.070)	1.017(0.997-1.038)	1.125(0.991-1.278)	1.126(0.962-1.318)
Respiratory mortality	0.957(0.886-1.033)	0.982(0.944-1.021)	0.874(0.677-1.127)	0.896(0.659-1.217)
Male	1.024(0.994-1.054)	1.008(0.991-1.025)	1.065(0.959-1.184)	1.045(0.917-1.190)
Female	1.036(0.999-1.074)	1.012(0.992-1.033)	1.100(0.965-1.253)	1.066(0.908-1.253)
≥65 years old	1.013(0.983-1.045)*	1.016(0.998-1.033)	1.094(0.981-1.221)	1.135(0.992-1.298)
<65 years old	**1.042(1.010-1.076)**	1.000(0.984-1.017)	1.042(0.937-1.160)	0.947(0.833-1.076)
Cold season				
Non-accidental mortality	**1.053(1.016-1.091)**	**1.043(1.025-1.062)**	**1.302(1.161-1.459)**	**1.387(1.214-1.585)**
Cardiovascular mortality	1.050(0.999-1.103)	**1.046(1.021-1.072)**	**1.318(1.123-1.546)**	**1.432(1.189-1.726)**
Respiratory mortality	1.072(0.960-1.196)	**1.070(1.014-1.129)**	**1.504(1.055-2.145)**	**1.717(1.135-2.597)**
Male	1.041(0.999-1.086)	**1.034(1.013-1.055)**	**1.228(1.074-1.405)**	**1.290(1.104-1.507)**
Female	**1.073(1.020-1.129)**	**1.059(1.033-1.086)**	**1.432(1.216-1.687)**	**1.560(1.289-1.889)**
⩾65 years old	1.020(0.976-1.066)*	**1.049(1.027-1.072)**	**1.294(1.123-1.491)**	**1.521(1.292-1.791)***
＜65 years old	**1.099(1.047-1.153)**	**1.035(1.011-1.060)**	**1.308(1.119-1.529)**	**1.221(1.016-1.468)**
Warm season				
Non-accidental mortality	0.981(0.945-1.019)	0.998(0.979-1.017)	0.970(0.857-1.098)	1.004(0.868-1.161)
Cardiovascular mortality	1.020(0.982-1.060)	1.003(0.984-1.022)	1.034(0.913-1.172)	0.999(0.863-1.156)
Respiratory mortality	0.993(0.898-1.097)	1.012(0.963-1.062)	1.051(0.761-1.452)	1.124(0.775-1.628)
Male	1.018(0.983-1.054)	1.006(0.988-1.023)	1.047(0.933-1.174)	1.030(0.900-1.178)
Female	1.007(0.960-1.057)	0.993(0.970-1.017)	0.974(0.832-1.140)	0.931(0.775-1.118)
⩾65 years old	1.035(0.996-1.077)	1.011(0.992-1.031)	1.095(0.963-1.244)	1.059(0.912-1.229)
＜65 years old	0.989(0.947-1.032)	0.989(0.968-1.010)	0.936(0.813-1.077)	0.915(0.777-1.078)

Bold values represent statistically significant results ($p < 0.05$).

* Significant results of Z tests for the difference between the two relative risks of subgroup analysis ($p < 0.05$).

The 99th percentile of DTR distribution (10.8 °C for the full year, 11.5 °C for the cold season and 8.8 °C for the warm season) with reference to the minimum relative risk in each subgroup.

Table S6. Relative risk (95% *CI*) of non-accidental mortality for extreme high DTR at single-day and cumulative lag periods with adjustment for co-pollutants.

**Pollutants**		**Lag 0**	**Lag 3**	**Lag 0–5**	**Lag 0–7**
$NO_2+PM_{10}+CO+SO_2+O_3$	Full year				
	Non-accidental mortality	1.012(0.991-1.033)	1.003(0.992-1.015)	1.029(0.955-1.108)	1.015(0.928-1.111)
	Cardiovascular mortality	1.019(0.983-1.057)	1.013(0.993-1.034)	1.089(0.957-1.240)	1.104(0.943-1.291)
	Respiratory mortality	0.944(0.862-1.034)	0.980(0.937-1.025)	0.853(0.632-1.152)	0.893(0.639-1.249)
	Male	1.002(0.992-1.012)	1.000(0.995-1.006)	1.004(0.968-1.041)	1.000(0.958-1.045)
	Female	1.025(0.988-1.062)	1.008(0.988-1.029)	1.068(0.937-1.216)	1.045(0.893-1.224)
	⩾65 years old	1.003(0.971-1.036)	1.012(0.994-1.030)	1.063(0.947-1.195)	1.110(0.966-1.276)
	<65 years old	0.967(0.925-1.010)	1.007(0.982-1.032)	1.001(0.855-1.173)	1.116(0.920-1.354)
	Cold season				
	Non-accidental mortality	1.025(0.987-1.065)	**1.032(1.013-1.052)**	**1.201(1.061-1.360)**	**1.300(1.128-1.498)**
	Cardiovascular mortality	1.013(0.959-1.069)	**1.035(1.008-1.063)**	**1.201(1.008-1.432)**	**1.353(1.107-1.654)**
	Respiratory mortality	1.047(0.934-1.174)	1.052(0.995-1.113)	1.351(0.929-1.964)	1.514(0.987-2.324)
	Male	1.007(0.961-1.054)	1.019(0.996-1.042)*	1.105(0.952-1.283)*	1.180(0.996-1.398)*
	Female	1.055(0.999-1.115)	**1.054(1.026-1.082)**	**1.372(1.149-1.639)**	**1.520(1.240-1.862)**
	⩾65 years old	1.003(0.956-1.053)	**1.041(1.017-1.066)**	**1.227(1.049-1.434)**	**1.450(1.216-1.730)***
	<65 years old	1.055(1.003-1.110)	1.020(0.995-1.046)	1.165(0.987-1.376)	1.121(0.925-1.359)
	Warm season				
	Non-accidental mortality	1.059(0.982-1.142)	1.017(0.979-1.057)	1.153(0.897-1.482)	1.085(0.809-1.454)
	Cardiovascular mortality	1.092(0.967-1.232)	1.023(0.962-1.088)	1.225(0.818-1.836)	1.104(0.688-1.771)
	Respiratory mortality	1.037(0.773-1.391)	1.080(0.934-1.248)	1.522(0.584-3.969)	1.950(0.649-5.856)
	Male	1.071(0.971-1.180)	1.027(0.978-1.079)	1.226(0.887-1.695)	1.176(0.806-1.716)
	Female	1.005(0.990-1.020)	1.000(0.992-1.008)	1.005(0.956-1.057)	0.994(0.937-1.054)
	⩾65 years old	**1.140(1.025-1.267)**	1.054(0.999-1.111)	**1.480(1.042-2.102)**	1.367(0.908-2.059)
	<65 years old	0.972(0.867-1.090)	0.975(0.920-1.033)	0.857(0.585-1.254)	0.819(0.526-1.276)
$NO_2+PM_{2.5}+CO+SO_2+O_3$	Full year				
	Non-accidental mortality	1.015(0.993-1.038)	1.004(0.992-1.017)	1.037(0.958-1.123)	1.021(0.927-1.124)
	Cardiovascular mortality	1.022(0.985-1.060)	1.014(0.993-1.035)	1.096(0.961-1.250)	1.106(0.943-1.297)
	Respiratory mortality	0.952(0.869-1.042)	0.983(0.940-1.028)	0.872(0.647-1.175)	0.908(0.650-1.267)

Male	1.004(0.991-1.018)	1.001(0.993-1.008)	1.008(0.959-1.058)	1.001(0.943-1.062)
Female	1.027(0.990-1.066)	1.010(0.989-1.031)	1.080(0.946-1.232)	1.059(0.902-1.243)
⩾65 years old	1.006(0.974-1.039)	1.013(0.995-1.032)	1.074(0.956-1.206)	1.121(0.975-1.289)
<65 years old	1.019(0.984-1.056)	0.989(0.971-1.007)	0.964(0.857-1.084)	0.878(0.768-1.004)
Cold season				
Non-accidental mortality	1.027(0.988-1.067)	**1.033(1.014-1.053)**	**1.209(1.068-1.369)**	**1.310(1.137-1.509)**
Cardiovascular mortality	1.015(0.962-1.071)	**1.035(1.008-1.063)**	**1.206(1.012-1.437)**	**1.352(1.107-1.652)**
Respiratory mortality	1.050(0.936-1.178)	1.055(0.997-1.116)	1.373(0.945-1.996)	**1.545(1.008-2.369)**
Male	1.009(0.964-1.056)	1.020(0.997-1.043)*	1.114(0.960-1.293)*	**1.189(1.004-1.408)***
Female	1.055(0.999-1.115)	**1.055(1.027-1.083)**	**1.377(1.153-1.644)**	**1.528(1.249-1.871)**
⩾65 years old	1.006(0.959-1.056)	**1.043(1.019-1.068)**	**1.244(1.064-1.454)**	**1.474(1.237-1.757)***
＜65 years old	1.054(1.002-1.109)	1.020(0.994-1.046)	1.162(0.984-1.371)	1.117(0.922-1.353)
Warm season				
Non-accidental mortality	1.058(0.981-1.141)	1.016(0.978-1.055)	1.145(0.890-1.472)	1.074(0.801-1.439)
Cardiovascular mortality	1.087(0.963-1.228)	1.022(0.961-1.087)	1.211(0.808-1.814)	1.093(0.681-1.754)
Respiratory mortality	1.040(0.776-1.395)	1.078(0.933-1.246)	1.518(0.583-3.956)	1.920(0.640-5.762)
Male	1.069(0.969-1.178)	1.025(0.975-1.077)	1.208(0.873-1.670)	1.150(0.788-1.679)
Female	1.005(0.990-1.020)	1.000(0.993-1.008)	1.006(0.957-1.058)	0.995(0.938-1.055)
⩾65 years old	**1.139(1.024-1.266)**	1.054(0.999-1.111)	**1.479(1.042-2.101)**	1.370(0.910-2.062)
＜65 years old	0.971(0.866-1.089)	0.972(0.917-1.030)	0.844(0.577-1.235)	0.799(0.513-1.245)

Bold values represent statistically significant results ($p < 0.05$).

* Significant results of Z tests for the difference between the two relative risks of subgroup analysis ($p < 0.05$).

The 99th percentile of DTR distribution (10.8 °C for the full year, 11.5 °C for the cold season and 8.8 °C for the warm season) with reference to the minimum relative risk in each subgroup.

Table S7. Relative risk (95% *CI*) of non-accidental mortality for extreme high DTR at single-day and cumulative lag periods with adjustment for apparent temperature.

Apparent Temperature		Lag 0	Lag 3	Lag 0–5	Lag 0–7
Df = 2	Full year				
	Non-accidental mortality	**1.032(1.009-1.056)**	1.011(0.997-1.024)	**1.088(1.001-1.182)**	1.057(0.954-1.172)
	Cardiovascular mortality	**1.040(1.004-1.076)**	1.019(0.999-1.040)	**1.143(1.008-1.297)**	1.134(0.970-1.326)
	Respiratory mortality	0.958(0.889-1.033)	0.986(0.948-1.025)	0.891(0.693-1.146)	0.924(0.683-1.251)
	Male	1.027(0.998-1.057)	1.009(0.992-1.025)	1.073(0.966-1.191)	1.045(0.919-1.189)
	Female	**1.039(1.002-1.076)**	1.013(0.993-1.034)	1.109(0.975-1.262)	1.074(0.916-1.261)
	⩾65 years old	1.017(0.987-1.048)*	**1.017(1.000-1.035)**	1.108(0.994-1.235)	**1.145(1.002-1.309)**
	<65 years old	**1.044(1.011-1.077)**	0.999(0.982-1.015)	1.036(0.932-1.151)	0.932(0.821-1.058)
	Cold season				
	Non-accidental mortality	**1.063(1.027-1.100)**	**1.046(1.029-1.064)**	**1.332(1.192-1.490)**	**1.404(1.231-1.602)**
	Cardiovascular mortality	**1.065(1.015-1.118)**	**1.052(1.027-1.078)**	**1.374(1.174-1.609)**	**1.480(1.228-1.783)**
	Respiratory mortality	1.078(0.969-1.198)	**1.072(1.017-1.131)**	**1.529(1.082-2.161)**	**1.738(1.157-2.612)**
	Male	**1.050(1.008-1.094)**	**1.037(1.016-1.058)**	**1.257(1.101-1.435)***	**1.309(1.120-1.529)***
	Female	**1.084(1.032-1.138)**	**1.062(1.036-1.088)**	**1.463(1.247-1.716)**	**1.572(1.301-1.898)**
	⩾65 years old	1.031(0.988-1.075)*	**1.052(1.030-1.075)**	**1.329(1.158-1.526)**	**1.545(1.315-1.815)***
	＜65 years old	**1.107(1.057-1.161)**	**1.038(1.014-1.062)**	**1.332(1.143-1.552)**	**1.232(1.027-1.479)**
	Warm season				
	Non-accidental mortality	1.017(0.991-1.044)	1.004(0.991-1.016)	1.035(0.952-1.126)	1.010(0.915-1.115)
	Cardiovascular mortality	1.038(0.991-1.087)	1.009(0.986-1.032)	1.083(0.931-1.258)	1.031(0.863-1.231)
	Respiratory mortality	0.993(0.897-1.099)	1.014(0.965-1.065)	1.063(0.767-1.472)	1.146(0.785-1.673)
	Male	1.021(0.986-1.058)	1.007(0.990-1.025)	1.059(0.944-1.188)	1.039(0.908-1.190)
	Female	1.014(0.967-1.064)	0.998(0.974-1.021)	1.002(0.859-1.169)	0.959(0.800-1.150)
	⩾65 years old	1.037(0.999-1.077)	1.012(0.994-1.032)	1.104(0.975-1.249)	1.069(0.924-1.237)
	＜65 years old	0.997(0.956-1.040)	0.993(0.973-1.014)	0.963(0.840-1.104)	0.941(0.801-1.105)
Df = 4	Full year				
	Non-accidental mortality	**1.032(1.008-1.056)**	1.010(0.997-1.024)	1.085(0.998-1.180)	1.054(0.951-1.169)
	Cardiovascular mortality	**1.037(1.002-1.074)**	1.017(0.997-1.038)	1.130(0.997-1.282)	1.118(0.956-1.307)

Respiratory mortality	0.961(0.891-1.036)	0.986(0.948-1.025)	0.895(0.696-1.152)	0.924(0.682-1.251)
Male	1.027(0.998-1.057)	1.008(0.992-1.025)	1.068(0.962-1.186)	1.040(0.914-1.183)
Female	**1.039(1.002-1.076)**	1.013(0.993-1.034)	1.111(0.976-1.264)	1.077(0.918-1.265)
≥65 years old	1.016(0.986-1.047)*	1.017(0.999-1.034)	1.103(0.989-1.230)	1.141(0.998-1.305)
<65 years old	**1.044(1.011-1.077)**	0.998(0.982-1.015)	1.034(0.930-1.149)	0.929(0.818-1.055)
Cold season				
Non-accidental mortality	**1.062(1.026-1.100)**	**1.047(1.029-1.065)**	**1.334(1.193-1.492)**	**1.410(1.236-1.609)**
Cardiovascular mortality	**1.066(1.015-1.119)**	**1.052(1.027-1.078)**	**1.373(1.172-1.608)**	**1.473(1.222-1.776)**
Respiratory mortality	1.076(0.968-1.197)	**1.073(1.018-1.131)**	**1.531(1.082-2.166)**	**1.749(1.162-2.633)**
Male	**1.050(1.008-1.094)**	**1.037(1.016-1.058)**	**1.257(1.101-1.435)***	**1.310(1.120-1.531)***
Female	**1.082(1.030-1.137)**	**1.063(1.037-1.089)**	**1.468(1.251-1.722)**	**1.589(1.316-1.920)**
≥65 years old	1.030(0.987-1.075)*	**1.053(1.031-1.075)**	**1.331(1.159-1.528)**	**1.551(1.320-1.823)***
<65 years old	**1.107(1.056-1.160)**	**1.038(1.014-1.063)**	**1.334(1.145-1.555)**	**1.238(1.031-1.487)**
Warm season				
Non-accidental mortality	1.014(0.991-1.038)	1.003(0.991-1.015)	1.028(0.952-1.111)	1.007(0.920-1.102)
Cardiovascular mortality	1.030(0.984-1.077)	1.006(0.983-1.029)	1.060(0.914-1.229)	1.015(0.853-1.207)
Respiratory mortality	0.999(0.901-1.108)	1.016(0.967-1.069)	1.084(0.778-1.512)	1.166(0.796-1.708)
Male	1.018(0.983-1.054)	1.006(0.988-1.023)	1.047(0.935-1.173)	1.030(0.902-1.177)
Female	1.015(0.967-1.066)	0.998(0.975-1.023)	1.007(0.861-1.179)	0.966(0.804-1.160)
≥65 years old	1.038(0.999-1.080)	1.013(0.994-1.032)	1.106(0.975-1.255)	1.070(0.924-1.241)
<65 years old	0.992(0.950-1.036)	0.991(0.970-1.013)	0.950(0.826-1.094)	0.932(0.792-1.098)

Bold values represent statistically significant results ($p < 0.05$).

* Significant results of Z tests for the difference between the two relative risks of subgroup analysis ($p < 0.05$).

The 99th percentile of DTR distribution (10.8 °C for the full year, 11.5 °C for the cold season and 8.8 °C for the warm season) with reference to the minimum relative risk in each subgroup.

Table S8. Relative risk (95% *CI*) of non-accidental mortality for extreme high DTR at single-day and cumulative lag periods with adjustment for lag periods.

		Lag 0	Lag3	Lag 0–5	Lag 0–7
Lag period: 5 days	Full year				
	Non-accidental mortality	**1.032(1.004-1.060)**	1.012(0.996-1.027)	**1.093(1.001-1.192)**	
	Cardiovascular mortality	**1.048(1.006-1.092)**	1.014(0.991-1.038)	1.125(0.985-1.284)	
	Respiratory mortality	1.024(0.945-1.109)	0.998(0.952-1.045)	1.011(0.777-1.315)	
	Male	1.019(0.985-1.054)	1.014(0.995-1.034)	1.095(0.980-1.222)	
	Female	**1.052(1.009-1.096)**	1.007(0.983-1.031)	1.087(0.951-1.242)	
	⩾65 years old	1.017(0.983-1.053)	1.017(0.997-1.036)	1.104(0.989-1.232)	
	<65 years old	**1.050(1.010-1.092)**	1.006(0.983-1.029)	1.081(0.953-1.227)	
	Cold season				
	Non-accidental mortality	**1.048(1.006-1.092)**	**1.056(1.034-1.078)**	**1.375(1.224-1.544)**	
	Cardiovascular mortality	**1.064(1.004-1.127))**	**1.057(1.027-1.088)**	**1.405(1.194-1.654)**	
	Respiratory mortality	1.072(0.943-1.218)	**1.082(1.014-1.154)**	**1.587(1.104-2.283)**	
	Male	1.029(0.980-1.080)	**1.048(1.023-1.074)**	**1.302(1.135-1.494)**	
	Female	**1.080(1.018-1.146)**	**1.068(1.036-1.100)**	**1.498(1.267-1.771)**	
	⩾65 years old	1.021(0.970-1.075)	**1.065(1.038-1.092)**	**1.398(1.213-1.611)**	
	<65 years old	**1.083(1.024-1.147)**	**1.045(1.015-1.075)**	**1.349(1.147-1.585)**	
	Warm season				
	Non-accidental mortality	0.985(0.970-0.999)	1.003(0.996-1.011)	1.000(0.957-1.045)	
	Cardiovascular mortality	1.019(0.957-1.084)	1.005(0.973-1.038)	1.042(0.869-1.250)	
	Respiratory mortality	0.957(0.847-1.081)	1.026(0.967-1.089)	1.090(0.773-1.538)	
	Male	1.030(0.991-1.071)	0.998(0.978-1.018)	1.019(0.910-1.142)	
	Female	1.041(0.985-1.101)	0.983(0.955-1.012)	0.957(0.813-1.125)	
	⩾65 years old	**1.059(1.012-1.108)**	1.002(0.979-1.025)	1.067(0.936-1.218)	
	<65 years old	1.007(0.958-1.059)	0.981(0.957-1.007)	0.916(0.792-1.059)	
Lag period: 14 days	Full year				
	Non-accidental mortality	**1.014(1.000-1.029)**	1.005(0.994-1.016)	1.039(0.972-1.111))	1.026(0.947-1.112)
	Cardiovascular mortality	1.018(0.995-1.041)	1.006(0.989-1.023)	1.049(0.944-1.166)	1.033(0.910-1.173)
	Respiratory mortality	1.009(0.951-1.070)	0.995(0.952-1.040)	0.984(0.743-1.302)	0.943(0.670-1.328)

Male	1.012(0.992-1.033)	1.004(0.989-1.019)	1.032(0.939-1.134)	1.020(0.910-1.144)
Female	1.017(0.994-1.041)	1.006(0.989-1.023)	1.047(0.941-1.166)	1.032(0.907-1.174)
≥65 years old	**1.022(1.000-1.045)**	1.013(0.997-1.030)	1.091(0.984-1.211)	1.098(0.968-1.246)
<65 years old	1.009(0.985-1.032)	0.997(0.980-1.015)	0.995(0.891-1.111)	0.963(0.843-1.101)
Cold season				
Non-accidental mortality	**1.059(1.034-1.084)**	**1.045(1.027-1.062)**	**1.317(1.184-1.465)**	**1.394(1.230-1.579)**
Cardiovascular mortality	**1.069(1.034-1.106)**	**1.051(1.027-1.076)**	**1.371(1.180-1.593)**	**1.454(1.219-1.735)**
Respiratory mortality	**1.084(1.008-1.166)**	**1.072(1.019-1.128)**	**1.537(1.111-2.127)**	**1.723(1.177-2.521)**
Male	**1.042(1.012-1.072)***	**1.033(1.012-1.054)***	**1.224(1.078-1.390)***	**1.280(1.102-1.486)***
Female	**1.085(1.049-1.122)**	**1.063(1.039-1.089)**	**1.476(1.269-1.716)**	**1.592(1.333-1.901)**
≥65 years old	**1.059(1.028-1.090)**	**1.050(1.029-1.072)**	**1.351(1.184-1.541)**	**1.461(1.252-1.705)**
<65 years old	**1.058(1.024-1.093)**	**1.038(1.014-1.061)**	**1.272(1.100-1.471)**	**1.309(1.104-1.553)**
Warm season				
Non-accidental mortality	0.987(0.962-1.013)	0.993(0.975-1.011)	0.953(0.849-1.070)	0.952(0.831-1.091)
Cardiovascular mortality	0.975(0.935-1.016)	0.989(0.961-1.018)	0.924(0.767-1.112)	0.936(0.753-1.163)
Respiratory mortality	1.012(0.940-1.089)	1.015(0.965-1.068)	1.092(0.789-1.513)	1.135(0.776-1.661)
Male	1.009(0.987-1.031)	1.006(0.991-1.022)	1.040(0.943-1.147)	1.045(0.932-1.172)
Female	1.008(0.977-1.040)	0.999(0.977-1.022)	1.004(0.869-1.160)	0.983(0.826-1.169)
≥65 years old	**1.021(1.002-1.040)**	1.012(0.999-1.026)	1.085(0.997-1.180)	1.091(0.988-1.204)
<65 years old	0.987(0.958-1.017)	0.990(0.970-1.011)	0.94(0.824-1.073)	0.93(0.796-1.086)

Bold values represent statistically significant results ($p < 0.05$).

* Significant results of Z tests for the difference between the two relative risks of subgroup analysis ($p < 0.05$).

The 99th percentile of DTR distribution (10.8 °C for the full year, 11.5 °C for the cold season and 8.8 °C for the warm season) with reference to the minimum relative risk in each subgroup.

Table S9. Relative risk (95% *CI*) of non-accidental mortality for extreme high TCN at single-day and cumulative lag periods by varying modeling choices.

	Modeling choices	Lag 0	Lag 3	Lag 0-5	Lag 0-7
Non-accidental mortality					
Full year		**1.086(1.058-1.115)**	**1.043(1.027-1.060)**	**1.342(1.213-1.486)**	**1.331(1.180-1.500)**
Cold season	Main model	**1.132(1.091-1.175)**	**1.071(1.050-1.093)**	**1.258(1.177-1.345)**	**1.611(1.384-1.876)**
Warm season		**1.063(1.023-1.106)**	**1.033(1.009-1.056)**	**1.248(1.077-1.445)**	**1.242(1.043-1.480)**
Full year	Df/year for time trend: 6	**1.091(1.063-1.121)**	**1.047(1.031-1.064)**	**1.375(1.243-1.522)**	**1.371(1.216-1.545)**
Cold season	Df for time trend: 2	**1.136(1.094-1.179)**	**1.077(1.055-1.098)**	**1.267(1.184-1.355)**	**1.682(1.444-1.961)**
Warm season		**1.064(1.024-1.106)**	**1.033(1.010-1.057)**	**1.254(1.083-1.451)**	**1.250(1.050-1.488)**
Full year	Df/year for time trend: 8	**1.086(1.057-1.115)**	**1.043(1.026-1.059)**	**1.337(1.209-1.480)**	**1.323(1.174-1.491)**
Cold season	Df for time trend: 4	**1.128(1.088-1.170)**	**1.067(1.047-1.089)**	**1.250(1.170-1.334)**	**1.566(1.348-1.820)**
Warm season		**1.064(1.023-1.106)**	**1.033(1.010-1.057)**	**1.252(1.081-1.450)**	**1.248(1.047-1.487)**
Full year		**1.069(1.040-1.098)**	**1.042(1.025-1.058)**	**1.311(1.193-1.440)**	
Cold season	Lag period: 5 days	**1.110(1.067-1.156)**	**1.062(1.039-1.086)**	**1.215(1.133-1.303)**	
Warm season		**1.059(1.015-1.105)**	**1.039(1.014-1.064)**	**1.281(1.111-1.478)**	
Full year		**1.080(1.050-1.111)**	**1.056(1.033-1.080)**	**1.418(1.235-1.629)**	**1.501(1.267-1.779)**
Cold season	Lag period: 14 days	**1.119(1.084-1.155)**	**1.090(1.065-1.116)**	**1.241(1.169-1.318)**	**1.923(1.610-2.298)**
Warm season		**1.073(1.038-1.109)**	**1.054(1.028-1.080)**	**1.393(1.191-1.630)**	**1.483(1.225-1.795)**
Cardiovascular mortality					
Full year		**1.117(1.073-1.163)**	**1.066(1.041-1.092)**	**1.538(1.317-1.795)**	**1.567(1.304-1.881)**
Cold season	Main model	**1.145(1.087-1.207)**	**1.087(1.056-1.118)**	**1.289(1.172-1.417)**	**1.814(1.462-2.250)**
Warm season		**1.112(1.045-1.183)**	**1.052(1.015-1.091)**	**1.434(1.136-1.811)**	**1.398(1.058-1.847)**
Full year	Df/year for time trend: 6	**1.123(1.079-1.169)**	**1.071(1.046-1.097)**	**1.586(1.359-1.850)**	**1.630(1.359-1.955)**
Cold season	Df for time trend: 2	**1.147(1.089-1.209)**	**1.090(1.060-1.121)**	**1.294(1.177-1.423)**	**1.865(1.504-2.311)**
Warm season		**1.112(1.045-1.183)**	**1.053(1.015-1.091)**	**1.438(1.140-1.814)**	**1.402(1.063-1.850)**
Full year	Df/year for time trend: 8	**1.116(1.072-1.162)**	**1.064(1.039-1.090)**	**1.525(1.306-1.781)**	**1.548(1.289-1.859)**
Cold season	Df for time trend: 4	**1.141(1.084-1.203)**	**1.083(1.053-1.114)**	**1.280(1.166-1.407)**	**1.767(1.426-2.190)**
Warm season		**1.114(1.047-1.185)**	**1.055(1.017-1.093)**	**1.452(1.151-1.833)**	**1.421(1.077-1.877)**
Full year	Lag period: 5 days	**1.118(1.073-1.165)**	**1.064(1.039-1.090)**	**1.523(-1.32-1.757)**	
Cold season		**1.146(1.083-1.213)**	**1.085(1.052-1.119)**	**1.289(1.169-1.422)**	

Warm season		**1.129(1.055-1.207)**	**1.042(1.003-1.082)**	**1.385(1.103-1.738)**	
Full year		**1.120(1.073-1.170)**	**1.086(1.050-1.123)**	**1.691(1.368-2.090)**	**1.853(1.430-2.402)**
Cold season	Lag period: 14 days	**1.152(1.101-1.205)**	**1.114(1.077-1.152)**	**1.312(1.204-1.430)**	**2.265(1.756-2.923)**
Warm season		**1.098(1.043-1.157)**	**1.069(1.027-1.112)**	**1.530(1.193-1.962)**	**1.638(1.210-2.216)**
Respiratory mortality					
Full year		1.071(0.976-1.175)	1.034(0.978-1.093)	1.265(0.885-1.809)	1.246(0.817-1.899)
Cold season	Main model	**1.178(1.048-1.324)**	**1.089(1.022-1.160)**	**1.352(1.095-1.669)**	**1.779(1.096-2.887)**
Warm season		1.081(0.933-1.253)	1.085(0.996-1.183)	1.627(0.933-2.838)	1.935(0.999-3.746)
Full year	Df/year for time trend: 6	1.072(0.977-1.176)	1.033(0.978-1.092)	1.263(0.886-1.800)	1.237(0.814-1.879)
Cold season	Df for time trend: 2	**1.191(1.057-1.342)**	**1.110(1.041-1.183)**	**1.386(1.118-1.718)**	**2.097(1.285-3.420)**
Warm season		1.088(0.940-1.261)	**1.092(1.002-1.190)**	1.692(0.973-2.941)	**2.034(1.055-3.921)**
Full year	Df/year for time trend: 8	1.070(0.975-1.174)	1.033(0.978-1.092)	1.261(0.882-1.804)	1.242(0.814-1.895)
Cold season	Df for time trend: 4	**1.179(1.049-1.326)**	**1.089(1.022-1.160)**	**1.354(1.096-1.673)**	**1.774(1.092-2.884)**
Warm season		1.080(0.932-1.252)	1.084(0.994-1.182)	1.617(0.927-2.821)	1.917(0.990-3.714)
Full year		1.052(0.958-1.156)	1.029(0.975-1.087)	1.216(0.876-1.688)	
Cold season	Lag period: 5 days	**1.179(1.041-1.336)**	1.027(0.958-1.101)	**1.328(1.069-1.649)**	
Warm season		1.031(0.879-1.209)	**1.162(1.064-1.269)**	**2.182(1.281-3.715)**	
Full year		1.001(0.907-1.106)	0.987(0.913-1.067)	0.936(0.575-1.524)	0.881(0.485-1.600)
Cold season	Lag period: 14 days	**1.168(1.058-1.289)**	**1.123(1.043-1.209)**	**1.347(1.115-1.626)**	**2.405(1.370-4.223)**
Warm season		1.107(0.978-1.253)	1.096(0.996-1.205)	1.749(0.963-3.177)	2.051(0.990-4.246)

Bold values represent statistically significant results ($p < 0.05$).

The 99th percentile of TCN distribution (3.3 °C for the full year, 3.9 °C for the cold season and 2.7 °C for the warm season) with a reference of 0 °C.

Table S10. Relative risk (95% *CI*) of non-accidental mortality for extreme high TCN at single-day and cumulative lag periods with adjustment for the DTR variable.

	**Lag 0**	**Lag 3**	**Lag 0–5**	**Lag 0–7**
Non-accidental mortality				
Full year	**1.085(1.055-1.115)**	**1.043(1.026-1.060)**	**1.336(1.202-1.486)**	**1.324(1.170-1.499)**
Cold season	**1.125(1.082-1.170)**	**1.068(1.046-1.090)**	**1.244(1.160-1.335)**	**1.575(1.344-1.845)**
Warm season	**1.069(1.027-1.112)**	**1.034(1.011-1.059)**	**1.266(1.091-1.470)**	**1.255(1.053-1.497)**
Cardiovascular mortality				
Full year	**1.113(1.067-1.161)**	**1.063(1.037-1.090)**	**1.513(1.287-1.778)**	**1.539(1.275-1.859)**
Cold season	**1.135(1.074-1.199)**	**1.081(1.049-1.113)**	**1.267(1.147-1.400)**	**1.746(1.395-2.186)**
Warm season	**1.114(1.046-1.187)**	**1.053(1.015-1.092)**	**1.442(1.139-1.826)**	**1.402(1.060-1.853)**
Respiratory mortality				
Full year	1.094(0.993-1.206)	1.045(0.987-1.107)	1.365(0.942-1.978)	1.341(0.871-2.067)
Cold season	**1.197(1.059-1.352)**	**1.096(1.026-1.171)**	**1.391(1.116-1.734)**	**1.855(1.125-3.059)**
Warm season	1.127(0.969-1.311)	**1.102(1.011-1.203)**	**1.835(1.045-3.222)**	**2.119(1.093-4.108)**

Bold values represent statistically significant results ($p < 0.05$).

The 99th percentile of TCN distribution (3.3 °C for the full year, 3.9 °C for the cold season and 2.7 °C for the warm season) with a reference of 0 °C.

Table S11. Relative risk (95% *CI*) of non-accidental mortality for extreme high TCN at single-day and cumulative lag periods with adjustment for co-pollutants.

**Pollutants**		**Lag 0**	**Lag 3**	**Lag 0–5**	**Lag 0–7**
NO_2+PM_{10}+CO +SO_2+O_3	Non-accidental mortality				
	Full year	**1.080(1.050-1.111)**	**1.041(1.024-1.059)**	**1.321(1.185-1.472)**	**1.314(1.158-1.492)**
	Cold season	**1.115(1.073-1.159)**	**1.064(1.042-1.086)**	**1.225(1.142-1.313)**	**1.537(1.314-1.796)**
	Warm season	**1.059(1.017-1.103)**	**1.031(1.007-1.056)**	**1.234(1.059-1.438)**	**1.231(1.026-1.475)**
	Cardiovascular mortality				
	Full year	**1.111(1.065-1.160)**	**1.064(1.037-1.091)**	**1.515(1.284-1.787)**	**1.548(1.276-1.879)**
	Cold season	**1.126(1.066-1.190)**	**1.077(1.046-1.109)**	**1.250(1.132-1.380)**	**1.712(1.371-2.136)**
	Warm season	**1.105(1.036-1.179)**	**1.050(1.011-1.091)**	**1.412(1.107-1.802)**	**1.384(1.037-1.848)**
	Respiratory mortality				
	Full year	1.081(0.980-1.193)	1.043(0.983-1.106)	1.332(0.911-1.947)	1.329(0.852-2.072)
	Cold season	**1.171(1.035-1.324)**	**1.089(1.020-1.163)**	**1.338(1.071-1.671)**	**1.797(1.092-2.957)**
	Warm season	1.121(0.962-1.306)	**1.104(1.009-1.208)**	**1.840(1.030-3.287)**	**2.166(1.092-4.297)**
NO_2+$PM_{2.5}$+CO +SO_2+O_3	Non-accidental mortality				
	Full year	**1.081(1.051-1.112)**	**1.042(1.024-1.059)**	**1.327(1.191-1.478)**	**1.320(1.163-1.498)**
	Cold season	**1.116(1.073-1.160)**	**1.063(1.042-1.086)**	**1.225(1.142-1.314)**	**1.534(1.312-1.793)**
	Warm season	**1.060(1.018-1.103)**	**1.031(1.007-1.056)**	**1.235(1.060-1.440)**	**1.233(1.028-1.478)**
	Cardiovascular mortality				
	Full year	**1.114(1.067-1.163)**	**1.066(1.039-1.093)**	**1.531(1.298-1.805)**	**1.568(1.293-1.902)**
	Cold season	**1.125(1.065-1.189)**	**1.077(1.046-1.109)**	**1.248(1.130-1.378)**	**1.706(1.367-2.130)**
	Warm season	**1.108(1.039-1.182)**	**1.052(1.013-1.093)**	**1.429(1.120-1.823)**	**1.401(1.048-1.871)**
	Respiratory mortality				
	Full year	1.085(0.984-1.198)	1.046(0.987-1.109)	1.360(0.931-1.986)	1.365(0.877-2.127)
	Cold season	**1.170(1.034-1.323)**	**1.090(1.021-1.164)**	**1.336(1.070-1.669)**	**1.812(1.101-2.983)**
	Warm season	1.112(0.955-1.296)	**1.102(1.007-1.206)**	**1.811(1.014-3.237)**	**2.156(1.085-4.284)**

Bold values represent statistically significant results ($p < 0.05$).

The 99th percentile of TCN distribution (3.3 °C for the full year, 3.9 °C for the cold season and 2.7 °C for the warm season) with a reference of 0 °C.

Table S12. Relative risk (95% *CI*) of non-accidental mortality for extreme high TCN at single-day and cumulative lag periods with adjustment for apparent temperature.

Apparent Temperature		Lag 0	Lag 3	Lag 0–5	Lag 0–7
Df = 2	Non-accidental mortality				
	Full year	**1.087(1.058-1.116)**	**1.044(1.027-1.060)**	**1.345(1.215-1.488)**	**1.332(1.182-1.502)**
	Cold season	**1.132(1.091-1.174)**	**1.071(1.050-1.093)**	**1.257(1.176-1.344)**	**1.611(1.383-1.875)**
	Warm season	**1.064(1.024-1.106)**	**1.033(1.010-1.057)**	**1.250(1.080-1.4470**	**1.244(1.044-1.482)**
	Cardiovascular mortality				
	Full year	**1.120(1.076-1.166)**	**1.067(1.042-1.093)**	**1.551(1.329-1.811)**	**1.579(1.315-1.896)**
	Cold season	**1.147(1.088-1.209)**	**1.087(1.057-1.118)**	**1.292(1.176-1.421)**	**1.817(1.464-2.255)**
	Warm season	**1.115(1.049-1.187)**	**1.054(1.016-1.093)**	**1.449(1.148-1.829)**	**1.409(1.067-1.861)**
	Respiratory mortality				
	Full year	1.067(0.973-1.171)	1.032(0.977-1.091)	1.251(0.876-1.788)	1.234(0.810-1.881)
	Cold season	**1.178(1.049-1.324)**	**1.089(1.022-1.160)**	**1.352(1.096-1.669)**	**1.779(1.097-2.886)**
	Warm season	1.075(0.928-1.244)	1.083(0.994-1.181)	1.602(0.921-2.789)	1.914(0.989-3.702)
Df = 4	Non-accidental mortality				
	Full year	**1.086(1.058-1.115)**	**1.043(1.027-1.060)**	**1.343(1.213-1.487)**	**1.331(1.180-1.501)**
	Cold season	**1.132(1.091-1.174)**	**1.071(1.050-1.093)**	**1.258(1.177-1.345)**	**1.615(1.387-1.880)**
	Warm season	**1.063(1.023-1.106)**	**1.032(1.009-1.056)**	**1.248(1.077-1.445)**	**1.241(1.041-1.479)**
	Cardiovascular mortality				
	Full year	**1.115(1.071-1.161)**	**1.065(1.040-1.091)**	**1.530(1.310-1.786)**	**1.562(-1.30-1.876)**
	Cold season	**1.145(1.087-1.207)**	**1.087(1.057-1.118)**	**1.289(1.172-1.417)**	**1.816(1.463-2.253)**
	Warm season	**1.111(1.044-1.183)**	**1.052(1.015-1.091)**	**1.433(1.135-1.810)**	**1.396(1.057-1.845)**
	Respiratory mortality				
	Full year	1.072(0.976-1.176)	1.034(0.978-1.093)	1.267(0.886-1.811)	1.246(0.817-1.900)
	Cold season	**1.176(1.047-1.322)**	**1.089(1.023-1.160)**	**1.349(1.092-1.665)**	**1.791(1.104-2.904)**
	Warm season	1.081(0.933-1.253)	1.085(0.995-1.183)	1.628(0.933-2.839)	1.936(0.999-3.749)

Bold values represent statistically significant results ($p < 0.05$).

The 99th percentile of TCN distribution (3.3 °C for the full year, 3.9 °C for the cold season and 2.7 °C for the warm season) with a reference of 0 °C.

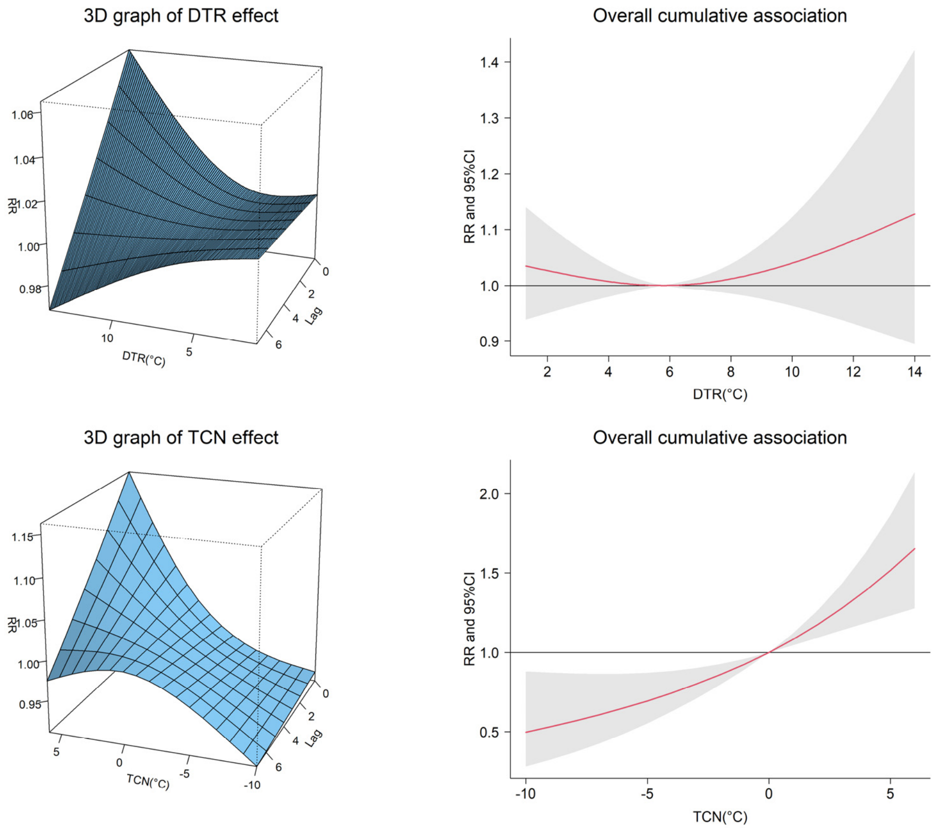

Figure S1. Exposure–response relationship between temperature change and risk of non-accidental mortality. Left panels: 3D plots of the exposure–lag-response risks. Right panels: overall exposure–response associations with diurnal temperature change (DTR) and temperature change between neighboring days (TCN).

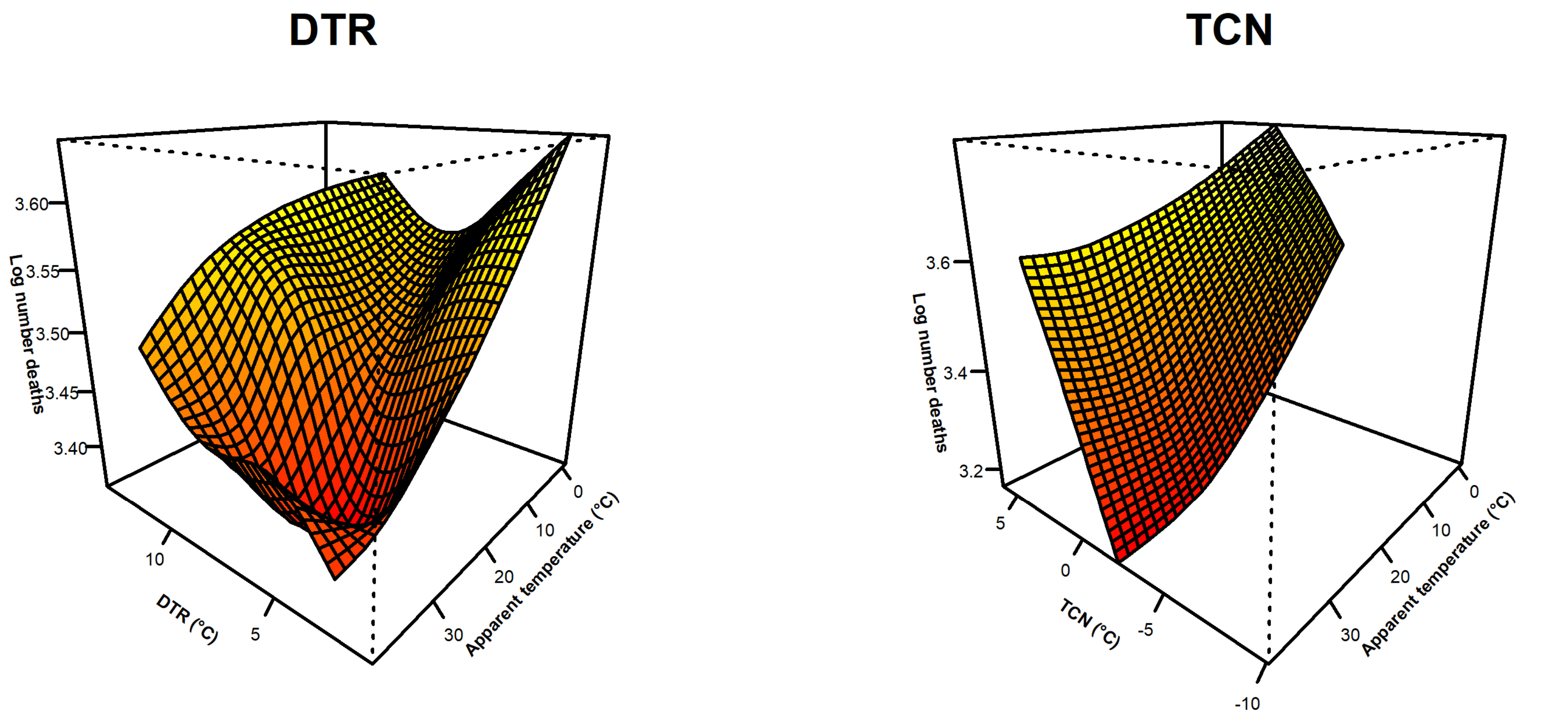

Figure S2. Bivariate response surface of apparent temperature and temperature change on non-accidental mortality (Left: lag 0–5 for DTR and lag 0 for AT; right: lag 0–5 for TCN and lag 0 for AT).
